# “Straw in the Clay Soil” Strategy: Anticarbon Corrosive Fluorine‐Decorated Graphene Nanoribbons@CNT Composite for Long‐Term PEMFC

**DOI:** 10.1002/advs.202402020

**Published:** 2024-09-19

**Authors:** Song Jin, JunHwa Kwon, Jong Min Lee, Ye‐Rim Kim, Justin Georg Albers, Young‐Woo Choi, Sung Mook Choi, KwangSup Eom, Min Ho Seo

**Affiliations:** ^1^ Energy & Environment Materials Research Division Korea Institute of Materials Science (KIMS) 797 Changwondaro Seongsangu Changwon Gyeongnam 51508 Republic of Korea; ^2^ School of Materials Science and Engineering Gwangju Institute of Science and Technology (GIST) 123 Cheomdangwagi‐ro Gwangju 61005 Republic of Korea; ^3^ Fuel Cell Research and Demonstration Center Hydrogen Energy Research Division Korea Institute of Energy Research (KIER) Jeollabuk‐do 56332 Republic of Korea; ^4^ Department of Nanotechnology Engineering Pukyong National University 45 Yongso‐ro, Nam‐gu Busan 48547 Republic of Korea; ^5^ Fraunhofer Institute for Manufacturing Technology and Advanced Materials IFAM Winterbergstrasse 28 01277 Dresden Germany; ^6^ Hydrogen Research Department Hydrogen Energy Research Division Korea Institute of Energy Research 152 Gajeong‐ro Yuseong‐gu Daejeon 34129 Republic of Korea; ^7^ Advanced Materials Engineering University of Science and Technology (UST) 113 Gwahangno Yuseong‐gu Daejeon 34113 Republic of Korea

**Keywords:** carbon corrosion, graphene nanoribbons, polymer electrolyte membrane fuel cell, density functional theory, transmission line model‐based impedance analysis

## Abstract

Carbon corrosion poses a significant challenge in polymer electrolyte membrane fuel cells (PEMFCs), leading to reduced cell performance due to catalyst layer degradation and catalyst detachment from electrodes. A promising approach to address this issue involves incorporating an anticorrosive carbon material into the oxygen reduction reaction (ORR) electrode, even in small quantities (≈3 wt% in electrode). Herein, the successful synthesis of fluorine‐doped graphene nanoribbons (F‐GNR) incorporated with graphitic carbon nanotubes (F‐GNR@CNT), demonstrating robust resistance to carbon corrosion is reported. By controlling the synthesis conditions using an exfoliation method, the properties of the composite are tailored. Electronic structural studies, employing density functional theory (DFT) calculations, to elucidate the roles of fluorine dopants and graphitic carbon nanotubes (CNTs) in mitigating carbon corrosion are conducted. Physicochemical and electrochemical characterization of F‐GNR@CNT reveal its effectiveness as a cathode additive at the single‐cell scale. The addition of F‐GNR@CNT to the Pt/C cathode improves durability by enhancing carbon corrosion resistance and water management, thus mitigating the flooding effect through tailored surface properties. Furthermore, advanced impedance analysis using a transmission line model is performed to gain insights into the internal resistance and capacitive properties of electrode structure.

## Graphical abstract

Anticarbon corrosion properties for oxygen reduction reaction cathodes in polymer electrolyte membrane fuel cell from the synergistic effect of fluorine doping and graphitic carbon nanotube in the F‐GNR@CNT composite. Long‐term performance maintenance in fuel cells validated by electrochemical *i*–V curves, density functional theory calculations, in situ electrochemical quartz crystal microbalance monitoring, and advanced electrochemical impedance spectroscopy analysis.

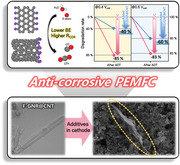

## 1 Introduction

Owing to the excessive use of fossil fuels and the myriad of environmental issues they cause, there has been a growing focus on mitigating global warming and achieving carbon neutrality. In response to these challenges, the enhancement of renewable energy technologies has emerged as a primary concern in contemporary society.^[^ ^1^ ^]^ Among these technologies, PEMFCs stand out as promising next‐generation renewable energy devices capable of replacing conventional fossil fuels, thus contributing to global warming mitigation and carbon neutrality.^[^ ^2^ ^]^ PEMFCs function by directly and efficiently converting electrochemical reactions from chemical energy into electrical energy, producing clean byproducts such as water and heat. Their appeal lies in a range of advantages, including high energy efficiency, environmentally friendly energy conversion, and low operating temperatures.^[^ ^3^ ^]^

Despite these advantages, the widespread adoption and commercialization of PEMFCs have been hindered by various scientific, durability, and cost‐effectiveness issues.^[^ ^2^, ^4^ ^]^ For PEMFCs to be widely commercialized, performance enhancements at high current densities are imperative. The catalyst layer (CL) plays a pivotal role in PEMFC systems as it hosts electrochemical reactions and governs maximum power density. A critical challenge within the CL lies in the sluggish catalytic ORR kinetics at the cathode, particularly problematic under high current densities.^[^ ^5^ ^]^ Additionally, adverse reactions worsen these challenges, as PEMFC systems operate in corrosive conditions that directly impact maintenance costs and lifespan. Typically, during repetitive start‐up/shut‐down cycles, the coexistence of fuel and oxygen gases at the anode generates a mixed potential, causing cathode potential strikes exceeding ≈1.4 V versus reversible hydrogen electrode (RHE), triggering destructive carbon corrosion reactions. Similarly, during fuel starvation conditions due to insufficient hydrogen supply during load fluctuations, the anode potential surpasses the cathode potential, leading to irreversible anode deterioration via electrochemical carbon corrosion reactions. Suppression of carbon corrosion is required because it can damage PEMFC performance by disrupting catalyst network, collapsing the electrode pore structure, and increasing the catalyst particle size. This leads to water flooding, which causes blockage of transfer channels and poor mass transport at high current densities.^[^ ^6^ ^]^ Therefore, a research strategy for maintaining CL structure and tailoring electrode surface properties, such as ensuring adequate wettability, is crucial.^[^ ^7^ ^]^ To overcome these issues, anticorrosive carbon‐based additives have been introduced into the ORR cathode.^[^ ^8^ ^]^ In our previous study, anticorrosive fluorine‐doped graphene nanoribbons (F‐GNRs) were developed and employed as additives in cathodes for long‐term PEMFC applications.^[^ ^9^ ^]^ These additives prevent carbon corrosion while maintaining CL structure and electrode surface properties.

Graphene nanoribbons (GNRs) have garnered interest as intriguing additive materials in cathodes owing to their extensive surface area and abundant edge sites, which are advantageous for tailoring their intrinsic properties through heteroatom doping engineering, unlike other carbon materials, such as Vulcan carbon, CNT, and graphene.^[^ ^10^ ^]^ However, the identical features that make GNRs advantageous can also be their drawback in terms of carbon corrosion, as they are prone to oxidation at numerous edge and defect sites.^[^ ^6^, ^11^ ^]^ To overcome this issue, heteroatom doping of carbon materials can modify their intrinsic properties, including durability and catalytic activity, through electronic structure modification.^[^ ^12^ ^]^ Among these dopants, fluorine stands out for its ability to enhance the durability of GNRs by forming the strongest covalent C–F bond among various single bonds, as summarized in Tables S1 and S2 (Supporting Information).^[^ ^13^ ^]^ The utilization of Pt/F‐GNR as an additive in a Pt/C cathode demonstrated improved stability of the membrane electrode assembly (MEA) in PEMFC during accelerated durability tests (ADTs) for carbon corrosion. Degradation rates of 25.0% and 6.24% were observed at 0.6 and 0.4 V, respectively, for the current density values. These voltages are dominant potential regions for activity kinetics and mass transport, respectively, compared to the 50.2% and 22.1% observed in a standard Pt/C‐based MEA. These results suggest that this improvement is attributed to the properties of F‐GNR, characterized by its large specific surface area and resistance to carbon corrosion.

In this study, we employed an effective strategy called “Straw in the Clay Soil” to enhance the durability of PEMFCs. By combining F‐GNRs with graphitic CNT composites (F‐GNR@CNT) as cathode additives, we achieved significantly improved corrosion resistance and enhanced electrical conductivity. These materials were applied as additives in the cathode, and we investigated the synergistic effects of both F‐GNR and graphitic multiwalled CNTs (MWCNTs) during practical PEMFC operation. This investigation involved a combination of DFT calculations and further electrochemical analysis using transmission line model (TLM)‐based advanced electrochemical impedance spectroscopy (EIS) analysis to elucidate the origin of the enhanced anticorrosive resistance in the CL structure.

The introduced strategy was found to improve the structural durability of the CL and aid in water management by mitigating carbon corrosion and preventing flooding effects during harsh cell operation conditions.

## 2 Results and Discussion

### 2.1 Theoretical Validation for the Synergistic Effect of F‐Doping and CNT Properties

**Figure 1:**
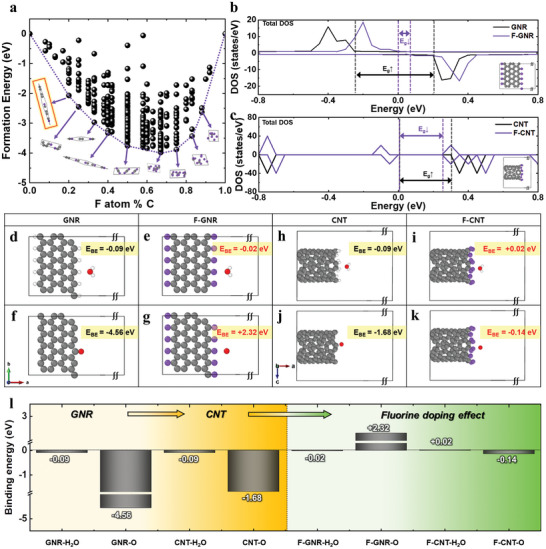
Various aspects of the DFT calculations and their implications for understanding the effect of fluorine doping and the presence of CNTs on carbon corrosion. a) Energy convex hull of 388 structure models is shown as a function of the doping level and the relative arrangement of fluorine and carbon atoms. The red box within the dashed purple line indicates a thermodynamically stable structure. b,c) Total DOS for GNR, F‐GNR, CNT, and F‐CNT. BE comparison of H_2_O molecule and O atom adsorbed to edge sites on d–g) GNR and F‐GNR, and h–k) CNT and F‐CNT, respectively. l) A summary highlighting the effect of CNT and fluorine doping through the BE comparison for H_2_O and O as carbon corrosion sources on the prepared models, including GNR, CNT, F‐GNR, and F‐CNT.

DFT calculations were conducted to validate the explicit durability roles and the synergistic effect of fluorine decorations on GNR and graphitic CNT composites for enhancing carbon corrosion as additive materials. The thermodynamically stable F‐GNR model structures were generated using the cluster expansion code from our previous study,^[^ ^9^ ^]^ as illustrated in **Figure** **1**a; and Figure S1 (Supporting Information).

The total density of states (DOS) was obtained, as depicted in Figure 1b; and Figure S2 (Supporting Information), providing valuable insights into the electronic bandgap, which is closely related to the electrical conductivity of materials, as measured by the powder resistivity measurement system (Figure S3 and Table S3, Supporting Information). The results indicate that F‐GNR@CNT exhibits better electrical conductivity (S cm^−1^) across various pressures (kgf) compared to other carbon materials. Notably, the synergistic effect of F doping and the graphitic CNT composite in F‐GNR@CNT results in higher conductivity than previously developed F‐GNRs in our group.

To compare the intrinsic carbon corrosion resistance of GNR, CNT, F‐GNR, and F‐CNT, the binding strengths of the H_2_O molecule and O atom were simulated as key guiding factors causing carbon corrosion according to reaction Equations (1) and (2) for the carbon corrosion mechanism^[^ ^7^, ^14^ ^]^

(1) $$C_{s}\left(s\right)+H_{2}O\left(l\right)↔C-O^{*}\left(s\right)+2H^{+}+2e^{-}$$

(2) $$C-O^{*}\left(s\right)+H_{2}O\left(l\right)↔CO_{2}\left(g\right)+2H^{+}+2e^{-}$$

In line with the trend of electron donation induced by fluorine doping and the subsequent decrease in bandgap (*E*_g_) observed in the DOS results, the binding energies (BEs) of H_2_O and O atoms on F‐GNR were weaker compared to those on GNR. Specifically, the BE of O atoms with F‐GNR exhibited a positive value of +2.32 eV (Figure 1d–g), indicating theoretically that O atoms do not bind to F‐GNR. Notably, F‐CNT displayed a similar characteristic with lower BEs with carbon corrosion sources (H_2_O and O) and intriguingly showed no binding with H_2_O molecules at positive values (+0.02 eV) (Figure 1h–k).

As summarized in Figure 1l, the robust anticorrosive properties of F‐GNR@CNT can be attributed to the synergistic effect of F‐GNR and F‐CNT, which exhibit positive values of O and H_2_O BEs, respectively. Through theoretical approaches via DFT calculations, the explicit durability roles and enhanced carbon resistance of F‐GNR@CNT can be investigated by comparing BEs with carbon corrosion sources.

### 2.2 Systematic Investigation for Physicochemical Properties of F‐GNR@CNT

**Figure 2:**
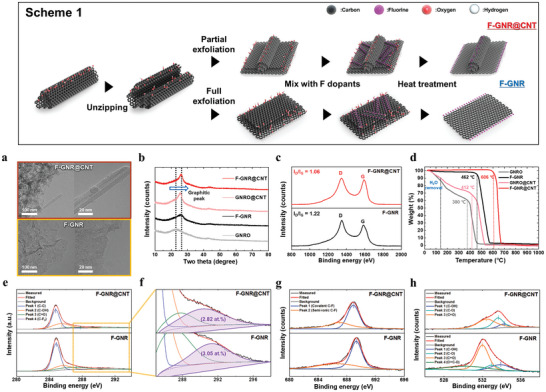
Various aspects of the synthesis process and characterization of F‐GNR and F‐GNR@CNT. Scheme 1) Synthetic process of F‐GNR and F‐GNR@CNT using the modified Hummer's method. a) TEM images showcasing the morphology of the synthesized materials. b) XRD patterns providing insights into the crystallinity of the prepared materials. c) Raman spectra indicating the presence of graphitic features and structural characteristics. d) TGA results depicting the thermal stability and decomposition behavior of the materials. e,f) F1s spectra and g) C1s spectra obtained from XPS analysis, revealing the presence of C–F bonds and providing quantitative information about the F doping content. h) O1s spectra from XPS analysis, indicating the presence of oxygen functional groups and defect sites in the materials.

In accordance with the intrinsic potential identified through DFT calculations to enhance carbon corrosion resistance, GNR‐derived materials were experimentally synthesized and subjected to physicochemical analysis to comprehend their properties. The synthesis process is illustrated in Scheme 1 of **Figure** **2**. F‐GNR@CNT or F‐GNR were synthesized by controlling the partial or full exfoliation states, respectively, using the modified Hummer's method followed by heat treatment for F doping. In the high‐resolution transmission electron microscopy (HR‐TEM) images (Figure 2a; and Figures S4 and S5, Supporting Information), F‐GNR@CNT exhibited a wing‐like morphology comprising 2D GNR and graphitic CNT, attributed to the intensively controlled partial exfoliation from the initial MWCNT during the synthetic process, contrasting with the fully exfoliated F‐GNRs. Additionally, the presence of F doping in F‐GNR@CNT and F‐GNR was evident from the high‐angle annular dark‐field scanning transmission electron microscopy (HAADF‐STEM) images with energy‐dispersive spectroscopy (EDS) mapping (Figures S6–S8, Supporting Information), which is performed postprocessing involved background removal and peak deconvolution using a multipolynomial model, parabolic order for background correction, and the Brown–Powell model using the ionization cross‐section model (Figure S9, Supporting Information). Interestingly, the graphitic peaks in GNRO@CNT and F‐GNR@CNT became sharper and positively shifted (Figure 2b), confirming the graphitic nature and revealing a lower *I*_D/G_ ratio of F‐GNR@CNT in Raman spectroscopy (Figure 2c), influenced by the presence of CNT. Furthermore, thermal gravimetric analysis (TGA) was conducted to validate the graphitic properties and structural stability of F‐GNR@CNT (Figure 2d). The order of thermal decomposition temperature was as follows: GNRO (≈380 °C) < GNRO@CNT (≈412 °C) < F‐GNR (≈462 °C) < F‐GNR@CNT (≈606 °C). Remarkably, GNRO@CNT exhibited a higher decomposition temperature than GNRO, indicating improved structural stability owing to the presence of the CNT composite. Particularly, F‐GNR@CNT displayed higher graphiticity and robust thermal stability, evidenced by its highest temperature denoted as T compared to the others, derived from the synergistic effects of CNT and F doping.

X‐ray photoelectron spectroscopy (XPS) analysis of F‐GNR and F‐GNR@CNT enabled quantitative determination of their elemental composition and chemical specificity. In the C1s spectra, the presence of the C–F bond was observed, indicating similar F doping contents of 2.82% and 3.05 at% for F‐GNR@CNT and F‐GNR, respectively. Additionally, both covalent and semi‐ionic C–F bonds were identified in the F1s spectra (Figure 2e–g).^[^ ^15^ ^]^ The existence of two types of C–F bonds in F‐GNR@CNT and F‐GNR can be attributed to the fluorination of the C–C bonds in GNRO or GNRO@CNT, typically involving two competing reactions. First, sp^3^‐hybridized carbon atoms form covalent C–F bonds by reacting with fluorine radicals to bond with the F atoms. These results in a reduction of the C–F bond length and the bond assumes a well‐defined orientation perpendicular to the graphitic planes under fluorination conditions. Second, sp^2^‐hybridized carbon atoms generate semi‐ionic C–F bonds through the reaction of fluorine radicals with GNRO or GNRO@CNT. It is well‐known that the transition from ionic to semi‐ionic to covalent C–F bonds leads to an increase in electron localization, which significantly affects carrier mobility.^[^ ^15^, ^16^ ^]^ F‐GNR exhibits significantly larger amounts of oxygen functional groups, such as C–OH (hydroxyl), C═O (carbonyl), and O═C–O (ester), indicating numerous defect sites and leading to rapid carbon corrosion compared to F‐GNR@CNT. Consequently, this provides direct evidence of the anticorrosive and stable states of F‐GNRs with CNT.

### 2.3 Electrochemical Carbon Corrosion Analysis with In Situ Electrochemical Quartz Crystal Microbalance (EQCM) Monitoring for Intrinsic Durability of F‐GNR@CNT

First, to understand the electrochemical property of F‐GNR and F‐GNR@CNT, the specific surface area of both materials was evaluated using cyclic voltammograms obtained at various scan rates ranging from 20 to 120 mV s^−1^. It revealed the presence of electric double‐layer capacitance. F‐GNR exhibited a higher double‐layer capacitance (1.86 mF cm^−2^), reflecting the electrochemical surface area of the carbon materials, compared to F‐GNR@CNT (1.28 mF cm^−2^) in Figure S10 (Supporting Information). These findings provide the evidence regarding the composite structure of F‐GNR and CNT, indicating that the incorporation of CNT, which has a lower surface area, results in a relatively reduced electrochemical surface area for F‐GNR@CNT compared to F‐GNR.

To understand the intrinsic electrochemical properties related to carbon corrosion resistance in F‐GNR and F‐GNR@CNT, electrochemical and quantitative analyses were conducted in a three‐electrode cell under half‐cell conditions. In situ EQCM analysis is time‐effective screening tool through real‐time monitoring mass change of carbon electrode caused by electro‐oxidation, such as the evolution of soluble organic carbon or CO_2(g)._^[^ ^17^ ^]^ To quantitatively and institutively monitor the carbon corrosion with mass changes by oxidiation current density increases of prepared carbon materials, such as F‐GNR, F‐GNR@CNT, and additional GNR sample, in situ EQCM was employed during the harsh condition on high potential at the constant 1.8 V for 20 h carbon corrosion. The relation between the change in resonance frequency and the mass change on the quartz crystal electrode was calculated by the following Sauerbrey Equations (3) and (4)^[^ ^18^ ^]^

(3) $$\Delta f=-\frac{2\Delta nf_{0}^{2}}{\sqrt{\mu_{q}\rho_{q}}}\cdot \frac{\Delta m}{A}$$

(4) $$\Delta m=-\frac{C}{n}\Delta f$$

where Δ*f* is the measured resonant frequency change (Hz), *n* is the fundamental mode of the crystal, Δ*f*_0_ is the resonant frequency of gold‐coated AT‐cut quartz crystal resonator (9 MHz), μ_q_ is the shear modulus of quartz (2.947 × 10^11^ g cm^−1^ s^−2^), ρ_q_ is the density of quartz (2.684 g cm^−3^), Δ*m* is the mass change, and *A* is the area of the gold disk coated on the crystal (0.196 cm^2^). In addition, the mass sensitivity constant (*C* = 5.45 ng cm^−2^), meaning a net change of 1 Hz corresponds to 5.45 ng of mass change on the crystal surface, is calculated by Equation (4).

**Figure 3:**
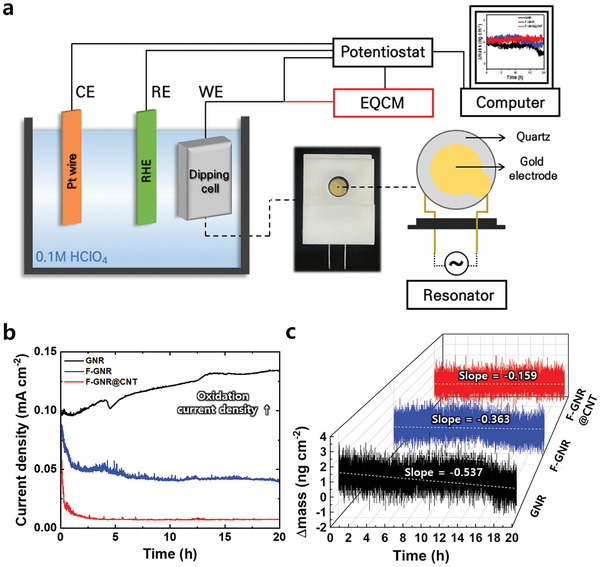
a) Set‐up schematic of the EQCM system. b) Current density per time at the constant 1.8 V for 20 h. c) Mass changes of GNR, F‐GNR, and F‐GNR@CNT with drop slope values recorded by EQCM under oxidation conditions.

As shown in **Figure** **3**b, GNR showed significantly positive current density incline, i.e., oxidation current increases, indicating its poor carbon corrosion resistance, while F‐GNR exhibited much lower current density change than GNR that it has the improved anticorrosion attributed by F doping effect. Interestingly, F‐GNR@CNT which has the synergistic effect of both F doping and graphitic CNT composites has nearly not changed at high potentials 1.8 V for 20 h. These trend of current density changes are clearly evident by the analysis results of EQCM in Figure 3c. According to the analysis results of EQCM, GNR has the steepest drop slop of mass change at −0.537 value, implying that serious structure collapse of the electrode causes significant carbon loss. In the case of F‐GNR, the drop lope of mass change is −0.363 value that it has little mass changes. Notably, F‐GNR@CNT showed the lowest mass changes at −0.159 for the drop slope value. These results indicate that F‐GNR@CNT as the additives could resist in the cathode during harsh carbon corrosion conditions at high potential (1.8 V), associated with lower carbon loss by minimizing the evolution of soluble organic carbon or CO_2(g)_ from the carbon electrodes caused by electro‐oxidation. In summary, the superior carbon corrosion resistance of F‐GNR@CNT was clearly demonstrated by quantifying amounts of carbon corrosion via the introduction of EQCM analysis, and even obtained the meaningful relationship between oxidation current density and mass drop as follows: 1) The order of oxidation current density increases: GNR > F‐GNR > F‐GNR@CNT; 2) The order of mass drop: GNR > F‐GNR > F‐GNR@CNT.

### 2.4 Practical MEA Evaluation for Applying Additives into Cathode in Single Cell Scale

**Figure 4:**
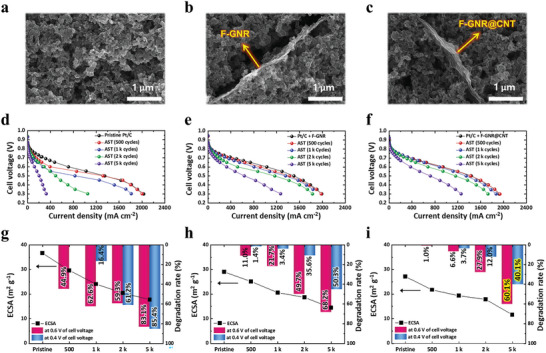
Top‐view SEM images illustrating the electrode configurations: a) Pristine Pt/C electrode, and electrodes incorporating F‐doped GNR and GNR@CNT additives for enhanced corrosion resistance: b) Pt/C+F‐GNR and c) Pt/C+F‐GNR@CNT. The electrochemical performance degradation and changes in ECSA during ASTs (DOE protocol on carbon corrosion condition) are depicted through d–f) polarization curves of Pt/C, Pt/C+F‐GNR, and Pt/C+F‐GNR@CNT, respectively. Quantitative analysis of ECSA values with decreasing performance rates measured at cell voltages of 0.6 and 0.4 V for g) Pt/C, h) Pt/C+F‐GNR, and i) Pt/C+F‐GNR@CNT during 5k ASTs.

Through the DFT calculations and half‐cell tests, the improved carbon corrosion resistance of F‐GNR@CNT highlighted by the hydrophobic properties of anhydrous C–F bonding and the thermodynamically unfavorable characteristics of CNT when exposed to carbon corrosion sources, such as H_2_O molecules and O atoms. This suggests the potential use of corrosion‐resistant electrodes for durable PEMFCs. To assess the feasibility of this approach, electrochemical analyses of full‐cell systems were conducted using electrodes comprising Pt/C catalysts and additives (F‐GNR and F‐GNR@CNT). **Figure** **4**a–c; and Figures S11–S13 (Supporting Information) depict top‐view images of well‐dispersed Vulcan carbon support (referred to as pristine Pt/C) and Pt/C electrodes containing 3 wt% of F‐GNR (Pt/C+F‐GNR) and F‐GNR@CNT (Pt/C+F‐GNR@CNT) additives, respectively. For single‐cell evaluation, the U.S. Department of Energy recommends an AST protocol^[^ ^19^ ^]^ for studying electrochemical carbon corrosion reactions, outlined in Figure S14 (Supporting Information). During AST processes, polarization (*i*–*V*) and CV curves were measured to assess the electrode performance and durability of fresh and degraded electrodes, as illustrated in Figure 4d–f; and Figure S15 (Supporting Information). In addition, the quantitative values regarding the output current at cell voltages of 0.6 V and 0.4 V, along with the electrochemical surface area (ECSA) of deteriorated electrodes, is presented in Table S4 (Supporting Information).

Under fresh conditions, the ECSA value of the pristine Pt/C electrode (36.68 m^2^ g^−1^) was found to be higher than that of fresh Pt/C+F‐GNR (29.01 m^2^ g^−1^) and Pt/C+F‐GNR@CNT (27.11 m^2^ g^−1^) electrodes. This difference is attributed to the hydrophobic properties of the additives, as confirmed by contact angle analysis (Figure S16, Supporting Information), resulting in reduced accessibility of proton ions to the Pt catalyst surface. However, there were no significant differences in initial catalytic activities, as evidenced by general polarization curves (Figure 4d–f) and similar charge transfer resistance (*R*_ct_) values (Figure S17, Supporting Information) of 1.0, 0.93, and 0.90 Ohm cm^2^ measured at a current density of 50 mA cm^−2^ for the pristine Pt/C, Pt/C+F‐GNR, and Pt/C+F‐GNR@CNT electrodes, respectively.

Nevertheless, as depicted in Figure 4g–i, the pristine Pt/C electrode exhibited drastic decay rates of the current density of 62.6% and 83.1% at 0.6 V cell voltage after 1k and 5k ASTs, respectively. In contrast, electrodes with F‐doped carbon additives (F‐GNR and F‐GNR@CNT) showed significantly lower degradation rates. Specifically, Pt/C+F–GNR exhibited degradation rates of 21.7% and 68.2%, while Pt/C+F‐GNR@CNT showed the lowest degradation rates of 6.6% and 60.1% at 0.6 V cell voltage after 1k and 5k ASTs, respectively. Similar degradation trends were observed at a cell voltage of 0.4 V after 5k ASTs, with Pt/C, Pt/C+F‐GNR, and Pt/C+F‐GNR electrodes exhibiting current decay rates of 85.4%, 50.3%, and 40.1%, respectively. Throughout ASTs, all open circuit voltages (OCVs) remained above 0.930 V in single‐cell systems, with a consistent level of reverse current density related to hydrogen crossover through the membrane electrolytes, indicating well‐preserved membrane stability. This degradation is attributed to the effects of the electrode, suggesting that the adoption of F dopants on carbon materials effectively suppresses carbon corrosion. Furthermore, the presence of graphitized CNT in F‐GNR@CNT further enhances corrosion resistance.

Interestingly, variations in ECSA did not directly correlate with degradation rates. As shown in Figure 4g–i, the pristine Pt/C exhibited a decrease in ECSA from 36.68 to 24.06 (34.4% loss) and 17.78 m^2^ g^−1^ (51.5% loss), accompanied by drastic performance deterioration. In contrast, Pt/C+F‐GNR@CNT showed ECSA decreases from 27.11 to 19.34 (28.7% loss) and 11.61 m^2^ g^−1^ (57.1% loss) with the lowest degradation rates after 1k and 5k ASTs, respectively. Given the significant difference in performance degradation rates, alongside no significant difference observed in ECSA decay rates between Pt/C and control groups with additives, as shown in Figure 4g–i, it is reasonable to assume that factors other than catalyst deterioration contribute to performance degradation in PEMFCs.

**Figure 5:**
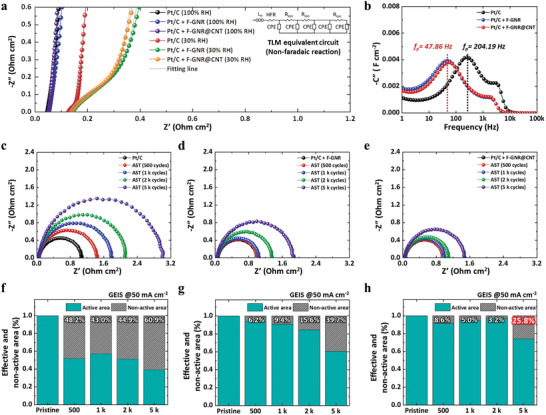
a) Electrochemical impedance results of pristine Pt/C, Pt/C + F‐GNR, and Pt/C + F‐GNR@CNT measured under non‐Faradaic conditions at 100% and 30% RH conditions with the TLM equivalent circuit, along with b) Imaginary capacitance conversion plots. c–e) Nyquist plots measured at 50 mA cm^−2^ of current density for the Faradaic reaction of Pt/C, Pt/C+F‐GNR, and Pt/C+F‐GNR@CNT electrodes, respectively. f–h) Rates of change in relaxation time values calculated by the correlation between capacitance and charge transfer resistance of Pt/C, Pt/C+F‐GNR, and Pt/C+F‐GNR@CNT electrodes, respectively, during 5k of ASTs.

This study primarily focused on the geometrical properties of porous electrodes since the capacitive feature of porous electrodes directly influences the three‐phase interface for the ORR and fuel cell performance. Therefore, to estimate the structural properties of degraded electrodes and identify the primary cause and origin of MEA performance decay, EIS analyses were conducted, as illustrated in **Figure** **5**.

### 2.5 EIS for Elucidating Resistive Phenomena in Electrode Structure

Prior to discussing electrode degradation behavior, we attempted to verify the influence of additives on water content in practical fuel cell systems. In Figure 5a, non‐Faradaic (capacitive) impedance analysis of fresh electrodes was conducted at 100% and 30% RH conditions, providing insights into the impacts of anhydrous C–F bonding on interfaces between ionomer and carbon support. All resistance and capacitance values of fresh and degraded electrodes were fitted using the TLM equivalent circuit depicted in Figure 5a. Variations in capacitive impedance results, along with detailed explanations of complex capacitance analysis, are shown in Figure S18 (Supporting Information). Numerical values for internal resistance and capacitive properties of fresh and degraded electrodes are summarized in Table S5 (Supporting Information). Impedance results measured at 100% RH conditions exhibit vertical lines of the general Randle circuit for capacitors due to negligible ionic resistance (*R*_ion_). Meanwhile, capacitive impedance plots measured at 30% RH conditions demonstrate distinctive resistance behaviors, presenting a straight line with a 45° slope corresponding to ionic resistance (*R*_ion_) in high‐frequency regions, as depicted in Figure 5a. Because of dehydrated ionomer conditions at 30% RH, both high‐frequency resistance (HFR) values, dominated by membrane resistance and electrical resistance of the electrode, and R_ion_ values increased, as shown in Figure 5a; and Table S5 (Supporting Information). Interestingly, it was observed that pristine Pt/C exhibits the *R*_ion_ value of 0.158 Ohm cm^2^, while Pt/C electrodes with ≈3 wt% additives (Pt/C+F‐GNR and Pt/C+F‐GNR@CNT) show more than quadrupled *R*_ion_ values of 0.765 and 0.720 Ohm cm^2^, respectively, suggesting that the introduction of fluorine dopants on carbon support increases surface hydrophobicity. The *R*_ion_ differences are clearly observed by the peak frequency (*f*_p_) in imaginary capacitance results shown in Figure 5b, indicating a higher peak frequency for pristine Pt/C (200.7 Hz) compared to Pt/C+F‐GNR and Pt/C+F‐GNR@CNT (≈47.9 Hz). Thus, it is speculated that weaker BE of additives with water molecules is a crucial cause of reduced corrosion rates, consistent with DFT predictions.

Impedance analyses were conducted to further clarify durable electrode conditions through F dopant and CNT introduction. Specifically, a TLM‐based impedance analysis was adopted to understand characteristics of a porous electrode during carbon support deformation, and Faradaic impedance measured at low current density of 50 mA cm^−2^ was used to evaluate activities of degraded MEA under exclusion condition of mass transfer interference by water formation and clogging. In TLM theory, real impedance (*Z*′) for Faradaic reaction and capacitance (*C*) of porous electrode can be expressed by the following Equations (5) and (6)^[^ ^20^ ^]^

(5) $$Z^{\prime }=\frac{R_{\mathrm{ion}}^{*}L}{3}+\frac{R_{\mathrm{ct}}^{*}}{XL}=\frac{R_{\mathrm{ion}}}{3}+R_{\mathrm{ct}}$$

(6) $$C=XLC_{\mathrm{dl}}^{*}$$

where $R_{\mathrm{ion}}^{*}$, $R_{\mathrm{ct}}^{*}$, and $C_{\mathrm{dl}}^{*}$ represent the ionic resistance per unit length, charge‐transfer resistance per unit area, and double‐layer capacitance per unit area, respectively; X and L indicate the pore circumference and depth factors in the cylindrical pore model. As depicted in these equations, the charge transfer resistance (*R*_ct_) and double‐layer capacitance (*C*_dl_) are inversely proportional to the structural factor (X L), indicating that the interfacial components of the porous electrode heavily influence the internal resistance of the water formation reaction and fuel cell performance. Therefore, variations in *R*_ct_ and *C*_dl_ values were traced to compare the degradation behaviors of the porous electrodes, as illustrated in Figure 5c,d; and Figure S18 (Supporting Information).

After 1k ASTs, both pristine Pt/C, Pt/C+F‐GNR, and Pt/C+F‐GNR@CNT electrodes exhibit capacitance increments owing to volume fractions resulting from electrode deformation caused by corrosion reactions; the *C*_dl_ value of pristine Pt/C increases from 0.0128 to 0.0130 F cm^−2^ (1.7%), that of Pt/C+F‐GNR increases from 0.0133 to 0.0139 F cm^−2^ (4.1%), and that of Pt/C+F‐GNR@CNT increases from 0.0123 to 0.0127 F cm^−2^ (2.7%), as shown in Figure S18 and Table S5 (Supporting Information). There were no significant differences between capacitance changes of the three electrodes; however, variations in *R*_ct_ for water formation reaction exhibit stark contrasts. *R*_ct_ of pristine Pt/C electrode increases from 1.0 to 1.73 Ohm cm^2^ (72.3%), that of Pt/C+F‐GNR increases from 0.93 to 0.98 Ohm cm^2^ (5.9%), and Pt/C+F‐GNR@CNT shows the lowest *R*_ct_ increment from 0.90 to 0.92 Ohm cm^2^ (2.5%) after 1k of ASTs, as shown in Figure 5c–e.

To evaluate different performance decay rates considering the geometrical characteristics, we focused on the specific relaxation times (*R*_ct_ x *C*_dl_), which reflect the internal resistance depending on the structural characteristics and variation in the degrading electrodes. Based on the relaxation time of the electrode deteriorated by the carbon‐corrosion‐induced ASTs, the relaxation time retention rate can be expressed as the following Equation (7)

(7) $$\mathrm{relaxation}\;\mathrm{time}\;\mathrm{retention}\;\mathrm{rate}=\frac{R_{\mathrm{ct},\;0}\;C_{\mathrm{dl},\;0}\;}{R_{\mathrm{ct},i}\;C_{\mathrm{dl},\;i}}=\frac{R_{\mathrm{ct},0}^{*}\;C_{\mathrm{dl},0}^{*}}{R_{\mathrm{ct},i}^{*}\;C_{\mathrm{dl},i}^{*}}$$

where 0 and i indicate the fresh and degraded electrode conditions after *i*‐cycles of AST. The physical meaning of the increase in $R_{\mathrm{ct},i}^{*}\;C_{\mathrm{dl},i}^{*}$, represented by retention rate below 1, is the lower catalytic rate per unit area and the increased capacitance per unit area due to electrode degradation. In Figure 5f–h, pristine Pt/C, Pt/C+F‐GNR, and Pt/C+F‐GNR@CNT electrodes indicate 57.0%, 90.6%, and 95.0% of relaxation time retention rates after 1k of ASTs, respectively. Similarly, the following degradation results also exhibit the lowest *R*_ct_ increment to 1.41 Ohm cm^2^ and highest relaxation time retention rate of 73.5% during 5k of ASTs, compared to deteriorated Pt/C (38.9%) and Pt/C+F‐GNR (60.4%) cases as shown in Figure 5f–h; and Table S5 (Supporting Information). To identify the major cause of the distinct performance decay rates and associated relaxation time variations, postmortem SEM analysis was performed. Most intuitively, the SEM images of the pristine and deteriorated Pt/C electrode after 5k ASTs clearly show the crumbled carbon structure, as shown in Figure S19a,d (Supporting Information). Corresponding to the drastic performance decay rates of the Pt/C electrode during ASTs, it is concluded that the high potential‐induced carbon corrosion reaction results in structural deformation and a decrease in the three‐phase condition for ORR, whose results are represented as the lowest relaxation time retention rate. In contrast, as shown in the SEM images of Pt/C+F‐GNR (Figure S19b,e, Supporting Information) and Pt/C+F‐GNR@CNT (Figure S19c,f, Supporting Information), there were no significant changes between the fresh and deteriorated electrodes, indicating relatively well‐maintained structural characteristics, which is indicated by the relatively high relaxation time retention rate of Pt/C with additives.

Accordingly, it could be concluded that the fluorine dopant on the carbon support (C–F bonding) and the lower BE of CNT with carbon corrosion sources (H_2_O molecules and O atoms), as predicted by DFT calculations and shown in the low humidity impedance results, would synergistically improve the structural durability of the porous electrode.

## 3 Conclusions

Anticorrosive carbon materials were successfully synthesized as cathode additives, specifically, F‐GNR@CNT composites containing hydrophobic C–F bonds, by tailoring the exfoliation state of the MWCNTs in a synthetic process. The F doping state and graphitic nature were confirmed through comprehensive physicochemical analyses. According to our DFT results in this study, the improved resistance to carbon corrosion in F‐GNR@CNT demonstrates that carbon corrosion sources, such as H_2_O molecules and O atoms, have much lower BEs than pristine GNR, owing to their high electron abundance and subsequent modification of the local electron density. Furthermore, our research revealed the distinct roles of F‐GNRs and F‐CNTs in terms of durability, as indicated by the positive BE values with carbon corrosion sources through the DFT calculations. This suggests that these carbon materials theoretically do not interact with H_2_O molecules or O atoms. The synergistic effect of both F doping and the graphitic nature of CNTs has been experimentally validated, demonstrating superior electrochemical carbon corrosion resistance with the lowest increases in oxidation current density and mass changes during carbon corrosion, compared to GNR and F‐GNR, as shown through in situ EQCM analysis.

Notably, our findings highlight the potential of F‐GNR@CNT as a cathode additive in practical PEMFC systems. This has been clearly elucidated through the carbon‐corrosion‐inducing AST condition, resulting in a robustly maintained well‐organized cathode electrode structure consisting of the catalyst, ionomer, and oxygen gas diffusion path for electro‐oxygen reduction, thereby overcoming the water‐flooding effect. In particular, advanced electrochemical interpretations of the electrode structure employing TLM‐based impedance analysis have provided valuable insights, including numerical values for the internal resistance and capacitive properties.

In conclusion, our study suggests that the anticorrosive F‐GNR@CNT holds promise as a cathode additive in durable PEMFC systems. This is achieved by preserving the porous electrode structure and mitigating water flooding because the synergistic effect of F doping and graphitic CNT features is clearly demonstrated through systematic combination approaches in both experiment and computation. We propose that our “Straw in the Clay soil strategy,” as called by our research group, may represent one of the crucial keys in the development of a robust and long‐lasting PEMFC system.

## 4 Experimental Section

### Computational Details

In this study, DFT calculations were conducted using the Vienna ab initio simulation package (VASP) to elucidate the synergistic effects of fluorine doping and the properties of CNTs in the anticorrosive F‐GNR@CNT composite, particularly in relation to adsorption with H_2_O and O as carbon corrosion sources.^[^ ^21^ ^]^ The ab initio electronic structure calculations employed the projector augmented wave pseudopotential method,^[^ ^22^ ^]^ utilizing the generalized gradient approximation for the exchange‐correlation energy and the revised Perdew–Burke–Ernzerhof functional.^[^ ^23^, ^24^ ^]^ To efficiently optimize the ionic and geometric structures, the Methfessel–Paxton smearing method was utilized.^[^ ^25^ ^]^ A plane‐wave basis set with a kinetic energy cut‐off of 520 eV was employed to ensure an accurate representation of valence electrons, while spin polarization was considered using the ‘ISPIN = 2’ calculation tag to analyze the electronic structure. High convergence and accuracy were ensured by employing a convergence criterion of 10^−5^ eV for the full ionic relaxation step, with maximum atomic forces limited to <0.01 eV Å^−1^. Additionally, to prevent interactions between the top and bottom of the unit cell box, a vacuum space of at least 20 Å along the z‐direction was implemented. Furthermore, the DFT‐D2 approach developed by Grimme et al.^[^ ^26^ ^]^ was utilized to determine the minimum formation energy of the F‐GNR structure.^[^ ^9^ ^]^ Geometrically relaxed structures were generated using the cluster expansion code, and the structure with the most thermodynamically stable formation energy was identified.^[^ ^27^, ^28^ ^]^ CNT models were generated using Materials Studio and geometrically optimized, with a gamma point mesh and (6 × 6 × 1) *k*‐points applied for geometry optimization. Hydrogen atoms were added to the edge sites of the GNR and CNT to maintain charge balance for accurate simulation. Importantly, the binding energies (BEs) of H_2_O adsorbed on the GNR and CNT were calculated as the initial step in the mechanism of carbon corrosion, followed by an investigation of the BEs of O adsorbed on GNR and CNT without hydrogen atoms. DOS calculations were performed to explore the electronic structural modifications on the prepared carbon materials, using Blöchl corrections based on the tetrahedron method.^[^ ^29^ ^]^

### Chemical and Materials

Carboxylic‐functionalized MWCNTs were acquired from Chengdu Organic Chemicals, with a purity exceeding 99.9 wt%, an outer diameter ranging from 10 to 20 nm, and lengths spanning from 5 to 30 µm. Chemicals and materials obtained from Sigma–Aldrich include phosphoric acid (H_3_PO_4_, 85 wt% in water), potassium permanganate (KMnO_4_), polytetrafluoroethylene preparation (PTFE, 60 wt% dispersion in water), perchloric acid (HClO_4_, 70%), and hydrogen peroxide solution (H_2_O_2_, 30 wt% in water). Sulfuric acid (H_2_SO_4_, 95%) and isopropyl alcohol (IPA, 99.5%) were sourced from Duksan Pure Chemicals, while a 1 N hydrochloric acid solution (HCl) was procured from Samchun Chemical. Potassium hydroxide flakes (KOH, 95%) were obtained from OCI Co., Ltd. Furthermore, 37.7 wt% Pt/C (TEC10V40E) was acquired from TANAKA Precious Metals, and Nafion ionomer solution (5 wt% in water, DE521) was procured from Ion Power. Deionized (DI) water with a resistivity of 18.2 µΩ was utilized in the experiments.

### Preparation of GNRO, GNRO@CNT, GNR, F‐GNR, and F‐GNR@CNT

In brief, graphene nanoribbon oxides (GNROs) and GNRO@CNT composites were synthesized using a modified Hummers and Tour method,^[^ ^30^ ^]^ as detailed in a procedure previously reported by our research group.^[^ ^9^ ^]^ The synthesis comprised the following steps: initially, 0.6 g of MWCNTs were immersed in 144 mL of sulfuric acid at room temperature for 1 h. Subsequently, 16 mL of H_3_PO_4_ solution was added as a secondary acid and stirred for 30 min. This step aimed to produce high‐quality GNROs by preventing the formation of surface defects. 3 g of KMnO_4_ was then gradually added, in an amount five times that of the MWCNTs, at a temperature below 5 °C for 1 h. Subsequently, the mixture was heated for 2 h, either at 60 or 35 °C, to fully or partially consume and react with KMnO_4_, respectively, thereby controlling the exfoliation and oxidation state. To lower kinetics and prevent explosive reactions that can occur when H_2_SO_4_, KMnO_4_, and water are present together, 480 mL of DI water was slowly added under vigorous magnetic stirring at a temperature below 5 °C. Under harsh acidic conditions, the MWCNTs were chemically unzipped and exfoliated. After stirring for 1 h, the solution was treated with 5 mL of H_2_O_2_ for ensuring the completion of reaction and eliminating excess of KMnO_4_ in the exothermic reaction. Subsequently, 600 mL of 10 vol% HCl solution was dispersed and stirred for 1 h to eliminate impurities and residual species. The solution was then filtered through a dialysis tube to remove any residual species. The GNRO and GNRO/CNT materials were obtained by freeze‐drying for 2 days. GNRO and GNRO/CNT were physically mixed with polytetrafluoroethylene (PTFE) powder at a 1:10 weight ratio. The sample was subjected to heat treatment at 1273 K for 1 h through a thermal shock/quench annealing process under an inert argon (Ar) atmosphere with a purity of 99.999%. Finally, F‐GNR@CNT and F‐GNR were obtained, in addition, reduced GNRO (referred to as GNR) was prepared to equal heat treatment condition without mixing PTFE powder.

### Preparation of Membrane Electrode Assembly (MEA) for PEMFC Operation

The MEAs were fabricated using the decal transfer method on a membrane, employing a Pt/C anode and three different types of cathode electrodes, including Pt/C and Pt/C with additives (≈3 wt% of F‐GNR and F‐GNR@CNT in total cathode amounts). Slurry inks were prepared by blending Pt/C powder, additives (F‐GNR, F‐GNR@CNT, or none), and a Nafion solution (5 wt%, Dupont) in a mixture of DI water and isopropyl alcohol (IPA) at a 1:1 volume ratio. The ratio of Nafion ionomer to carbon is fixed at 0.6 in the prepared slurry inks and the targeted dispersion amount of Nafion ionomer is 25 wt% of the total electrode solid. The mixture was subjected to ultrasonication for 12 h, and the dispersion was processed using equipment (SDS80, EXAKT 50 I) with a 10 µm gap between each of the three rolls. The resulting slurry was coated onto a polyimide film with a thickness of 100 µm, acting as a decal substrate using the Doctor blade coater. The coated electrode was then dried in an oven at 80 °C for 3 h under a nitrogen (N_2_)‐saturated atmosphere. Subsequently, it was hot‐pressed onto a Nafion 211 membrane, aligned with the anode electrode, at 50 bar and 110 °C for 10 min. After cold pressing for 10 min, the decal film was peeled off. The Pt metal loadings in the cathode and anode were ≈0.25 and 0.2 mg cm^−2^, respectively. The prepared MEAs were assembled using a gas diffusion layer (GDL, 39BB, SGL), Teflon gasket films, and a bipolar plate (BP) with four serpentine flow channels. The prepared MEAs were evaluated using a PEMFC test station (CNL Energy Co., Ltd.) with a 9 cm^2^ (3 cm × 3 cm) single cell. Prior to the electrode durability tests, the prepared single cells underwent activation at a 0.4 V cell voltage for 24 h.

### Physicochemical Characterizations

Surface elemental composition and chemical states were examined using XPS with a K‐alpha instrument from Thermo Fisher Scientific. Peak deconvolution was carried out using XPSPEAK software, and background calibration was performed using the Shirley method. The BE of C1s was adjusted to 284.2 eV to correspond to the C–C binding site in sp^2^ carbon. HAADF‐STEM with EDS mapping was employed to investigate the morphology and presence of fluorine in the carbon materials, utilizing a Titan Themis Z instrument from Thermo Scientific. Powder X‐ray diffraction (XRD) was conducted using an Empyrean instrument from Malvern Panalytical with Cu Kα sources at 1.5405 Å. The analysis involved a scan range from 10° to 80°, a scan speed of 5° min^−1^, and a scan step of 0.02°. Electrical conductivities of the prepared carbon materials were investigated using powder resistivity measurement systems (HPRM‐FA2, HANTECH). The ratio of defect and graphitic intensity (*I*_D/G_) for understanding the graphiticity of F‐GNR and F‐GNR@CNT is analyzed through Raman spectroscopy (LabRAM HR Evolution, Horiba). Thermal stability of prepared carbon materials, such as GNRO, GNRO@CNT, F‐GNR, and F‐GNR@CNT was assessed via TGA performed on a TGA‐50 instrument from Shimadzu Corp. The analysis involved heating the samples to 1000 °C at a rate of 10 °C min^−1^ under an air atmosphere. Scanning electron microscopy (SEM) was utilized to analyze the initial and postmortem structures of the CL of the pristine Pt/C cathode with and without additives, such as F‐GNR@CNT and F‐GNR. Contact angle analysis was conducted using Surfaceware software to measure the hydrophilicity and hydrophobicity of the prepared cathode.

### Electrochemical Measurements

In the half‐cell electrochemical evaluation, a potentiostat (ZIVE MP2A, PINE Research Products) and rotator were employed in a three‐electrode cell. The working, counter, and reference electrodes comprised glassy carbon (0.196 cm^2^), Pt wire, and a reversible hydrogen electrode (RHE), respectively. Prior to the experiment, the glassy carbon electrode underwent thorough polishing with 0.5 and 0.05 µm alumina dispersion, followed by sonication in ethanol and DI water multiple times for 1 min each.

The catalyst ink was prepared by combining optimized proportions of 5 wt% Nafion solution with 1.8 mg of catalysts. Subsequently, 0.5 mL of the solution, mixed with isopropyl alcohol, was dispersed into DI water at a 3:1 ratio and sonicated for several minutes below 35 °C. The loading of F‐GNR@CNT and F‐GNR was targeted at 100 µg cm^−2^.

To understand the specific surface area of the prepared carbon materials, cyclic voltammetry (CV) was performed under an N_2_ (purity: 99.999%)‐saturated 0.1 m HClO_4_ solution at 0.05–0.8 V with various scan rates ranging from 20 to 120 mV s^−1^.

EQCM was employed to evaluate corrosion behavior and quantify the degree of carbon corrosion. All EQCM experiments were performed in an QCA922A equipment (SEIKO EG&G, Japan) and a standard three‐electrode cell with gold‐coated AT‐cut quartz crystal resonator into dipping EQCM cell as the working electrode, RHE as the reference electrode, and a platinum wire as the counter electrode, was used in the EQCM experiment. The catalyst ink was the same as mentioned above in the RDE test, and it was deposited by the drop‐casting procedure onto a gold‐quartz crystal resonator. The loading of all carbon materials was targeted at 100 µg cm^−2^. All tests were carried out at room temperature at in a 0.1 m HClO_4_ solution under the harsh condition (high potential at the constant 1.8 V for 20 h) for carbon corrosion.

Moreover, the performance of the MEA was evaluated using an EIS potentiostat (BioLogic Science Instruments HCP‐803) to validate the effect of employing F‐GNR@CNT and F‐GNR as cathode additives in a single cell (CNL energy), which comprised the MEA with an active electrode area of 9 cm^2^. Polarization curves (*i*–*V*) were measured using the potential sweep method at a cell temperature of 70 °C under 100% relative humidity (RH) condition without any back pressure to evaluate the performances of degraded MEA in the single‐cell systems. During *i*–*V* curve measurements, 100/340 sccm of high‐purity H_2_ (99.999%) and air gas were fed to the anode and cathode, respectively, with the flow rates automatically increasing following the stoichiometry ratios(SRs) of 1.5 (fuel)/2.0 (air) as output currents exceeded the current density of 400 mA cm^−2^.

The ECSA was estimated by measuring CV curves with a potential sweep rate of 50 mV s^−1^ between 0.05 and 1.2 V versus RHE after purging H_2_ and N_2_ gases into the anode and cathode, respectively. The ECSA of the prepared MEAs was calculated using Equation (8)^[^ ^31^ ^]^

(8) $$ECSA=\frac{Q_{H}}{\left[Pt\right]*210}$$

where *Q*_H_ is the charge of hydrogen desorption on the Pt surface (µC cm^−2^) in the hydrogen region (0.075–0.40 V) of the CV curves, 210 µC cm^−2^ is the electrical charge with monolayer adsorption of hydrogen on the Pt surface, and [Pt] is the loading of Pt in cathode electrode that conclude Pt/C and GNR additives. Faradaic impedance analyses were conducted using a potentiostat (HCP‐803, Biologic) at a current density of 50 mA cm^−2^ in galvanostatic EIS mode, employing a 10% sinusoidal current amplitude at frequencies ranging from 10 kHz to 100 mHz. Non‐Faradaic (capacitive) impedance analyses were carried out at 0.4 V versus RHE in potentiostatic EIS mode with a 10 mV sinusoidal potential amplitude between 10 kHz and 30 MHz under 100% and 30% RH conditions, respectively. Carbon corrosion‐inducing ASTs at a cell temperature of 70 °C under 100% RH condition were performed using the triangular potential sweep method between 1.0 and 1.5 V versus RHE at a scan rate of 500 mV s^−1^. Each potential cycling test lasted for 1 second per cycle, and electrochemical analyses were conducted after the activation process (for the pristine state) and in the middle of 500, 1 k, 2 k, and 5 k cycles to evaluate the degraded MEA condition. Numerical values for output current at 0.6 and 0.4 V cell voltage, and ECSA of degraded electrodes, are summarized in Table S3 (Supporting Information).

## Conflict of Interest

The authors declare no conflict of interest.

## Author Contributions

S.J. and J.K. contributed equally to this work as co‐first authors. M.H.S., K.E., and S.M.C. conceived and supervised the study. S.J. conducted the material design, electrochemical tests (half‐cell), EQCM analysis, and DFT calculations. J.K. contributed to the electrochemical tests (single cell) and impedance analysis. Data curation and experimental analyses were supported by J.M.L., Y.‐R.K., J.G.A., and Y.‐W.C. This manuscript was written with contributions from all authors.

## Supporting information

Supporting Information

## Data Availability

The data that support the findings of this study are available from the corresponding author upon reasonable request.
