# Supplementary material for: “Straw in the Clay Soil” Strategy: Anticarbon Corrosive Fluorine‐Decorated Graphene Nanoribbons@CNT Composite for Long‐Term PEMFC
Source: Adv Sci (Weinh). 2024 Sep 19;11(45):2402020. doi: 10.1002/advs.202402020 (PMC11615808; doi:10.1002/advs.202402020)
Supplement: Supplementary file 1 — Supporting Information [file ADVS-11-2402020-s001.docx]

**Supporting Information**

**“Straw in the Clay Soil” Strategy: Anti-carbon corrosive fluorine-decorated Graphene nanoribbons@CNT composite for long-term PEMFC**

Song Jin^†^, JunHwa Kwon^†^, Jong Min Lee, Ye-Rim Kim, Justin Georg Albers, Young-Woo Choi, Sung Mook Choi*, KwangSup Eom*, and Min Ho Seo*

S. Jin, S. M. Choi Department of Hydrogen Energy Materials, Surface Technology Division, Korea Institute of Materials Science (KIMS) 797 Changwondaro, Seongsangu, Changwon, Gyeongnam 51508, Republic of Korea E-mail: akyzaky@kims.re.kr

S. Jin, J. Kwon, K. Eom School of Materials Science and Engineering, Gwangju Institute of Science and Technology (GIST), 261 Cheomdan-gwagiro, Gwangju 500-712, Republic of Korea E-mail: [keom@gist.ac.kr](mailto:keom@gist.ac.kr)

J. M. Lee

Fuel Cell Research and Demonstration Center, Hydrogen Energy Research Division, Korea Institute of Energy Research (KIER), Jeollabuk-do 56332, Republic of Korea

J. G. Albers Fraunhofer Institute for Manufacturing Technology and Advanced Materials IFAM, Winterbergstrasse 28, 01277 Dresden, Germany

Y. –W. Choi Hydrogen Research Department, Hydrogen Energy Research Division, Korea Institute of Energy Research, 152 Gajeong-ro, Yuseong-gu, Daejeon, 34129 Republic of Korea, Republic of Korea

S. M. Choi Advanced Materials Engineering, University of Science and Technology (UST), 113 Gwahangno, Yuseong-gu, Daejeon 34113, Republic of Korea

Y.-R. Kim, M. H. Seo Department of Nanotechnology Engineering, Pukyong National University, 45 Yongso-ro, Nam-gu, Busan 48547, Republic of Korea E-mail: [foifrit@pknu.ac.kr](mailto:foifrit@pknu.ac.kr)

† These authors contributed equally to this work.


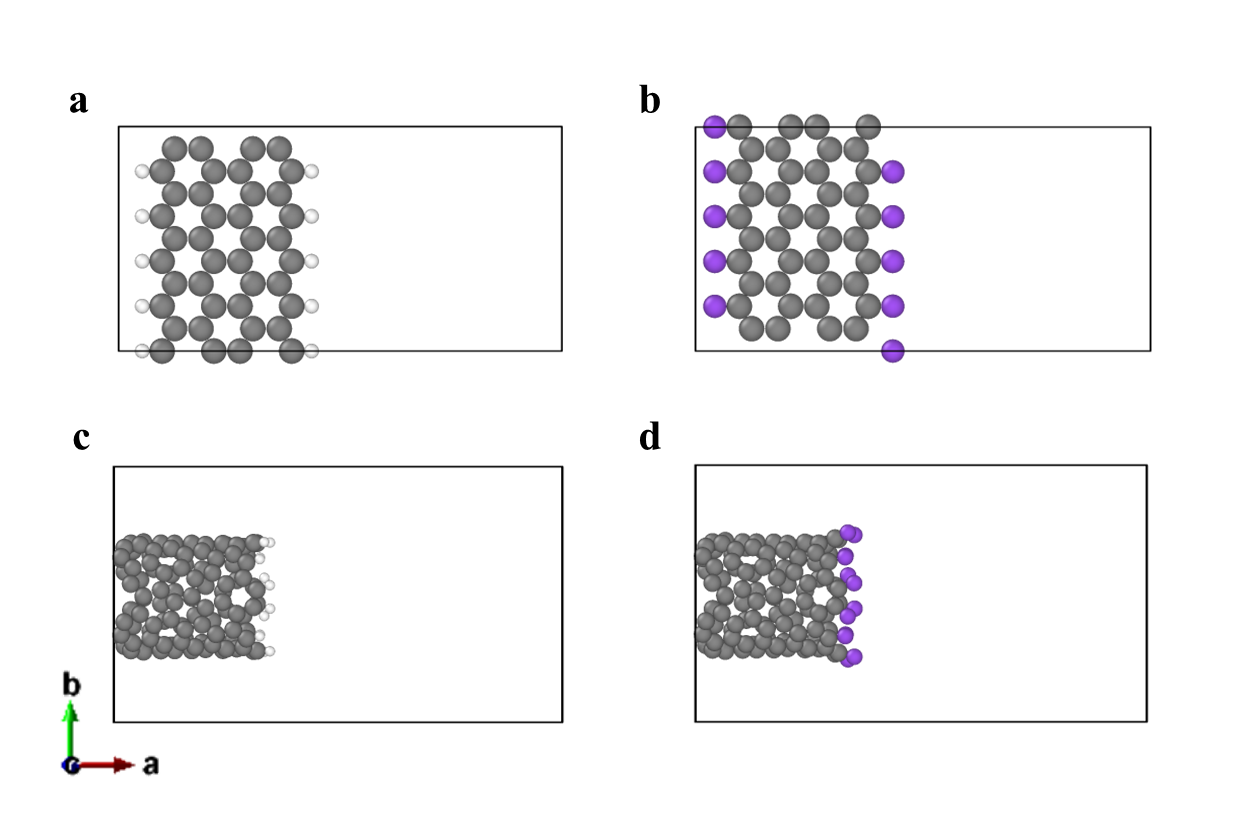


**Figure S1** Defined pristine model structures of (a) GNR, (b) F-GNR, (c) CNT, and (d) F-CNT.


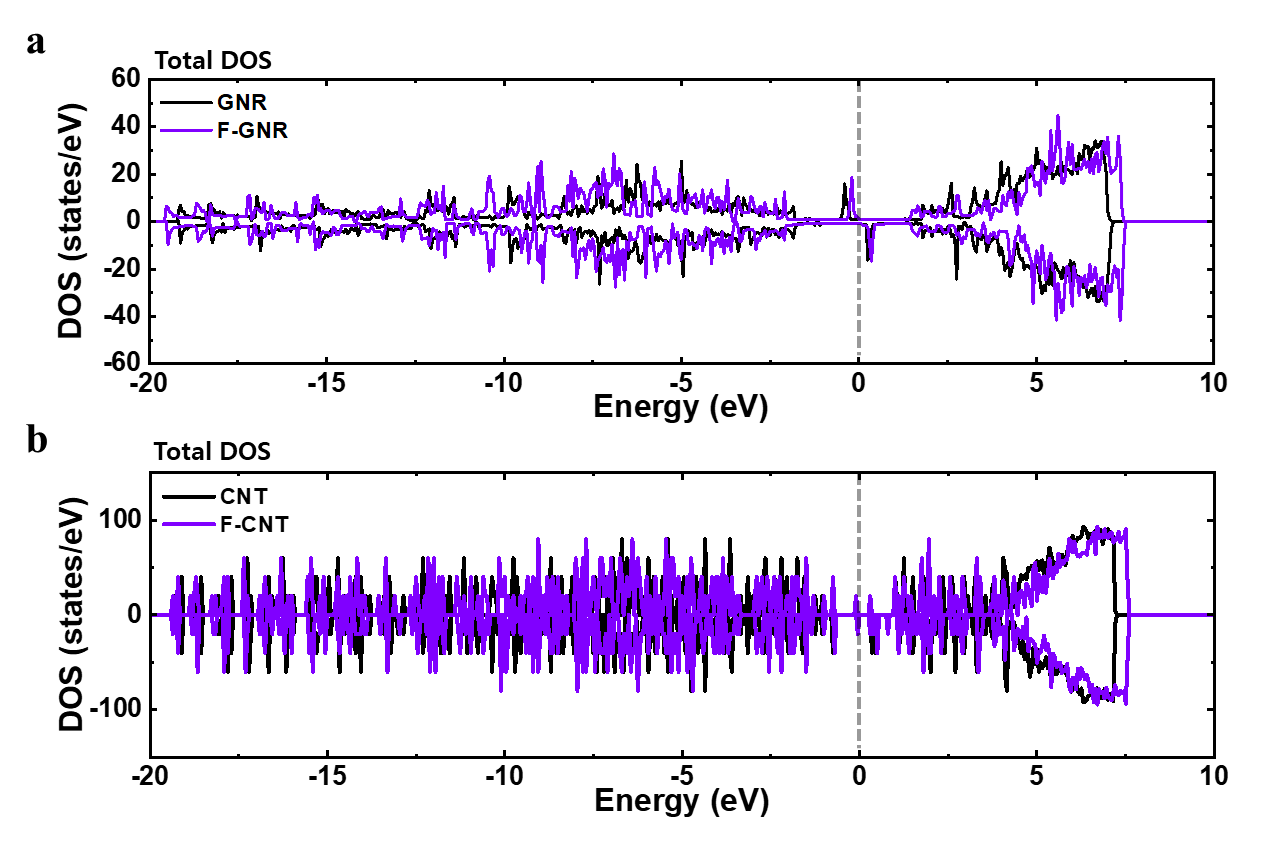


**Figure S2** Total DOS of (a) GNR and F-GNR, and (b) CNT and F-CNT in total range.

**Figure S3** Electrical conductivity comparison for Vulcan carbon, GNRO, F-GNR, GNRO@CNT, and F-GNR@CNT measured by powder resistivity measurement system.


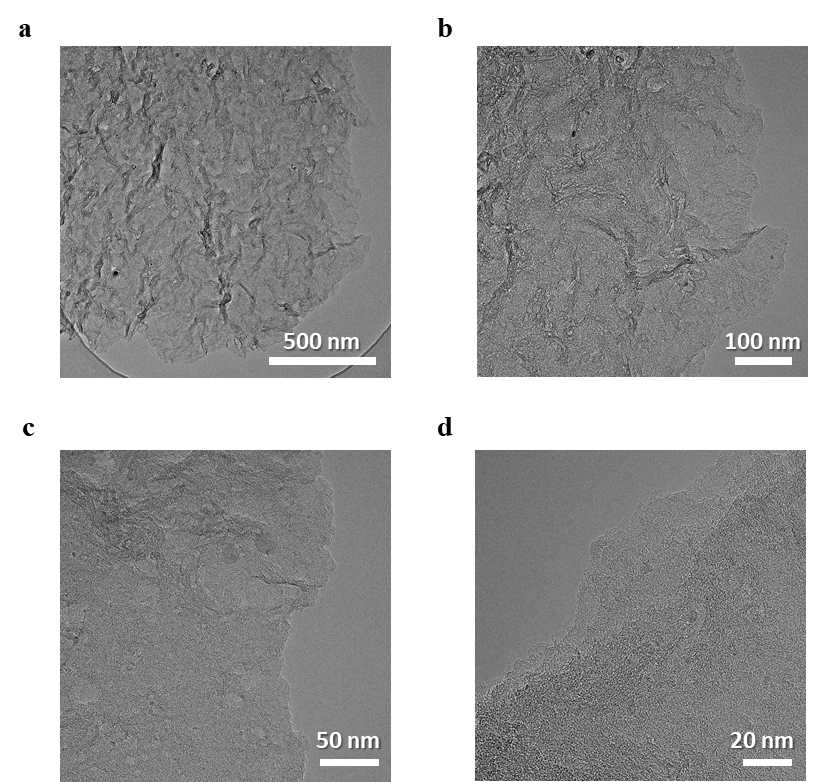


**Figure S4** TEM images from low-resolution to high-resolution F-GNR in order.


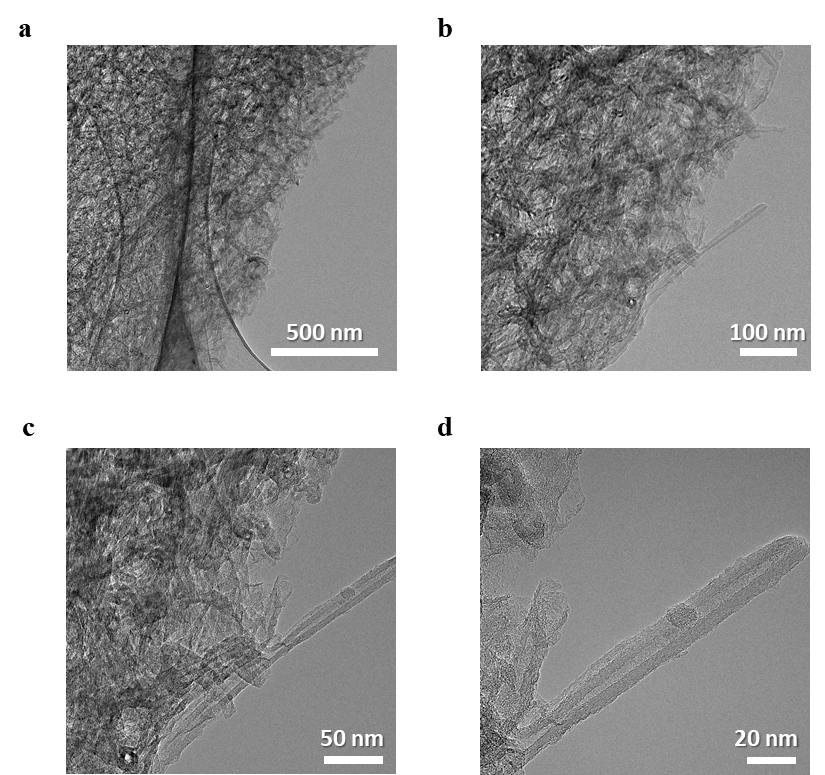


**Figure S5** TEM images from low-resolution to high-resolution F-GNR@CNT in order.


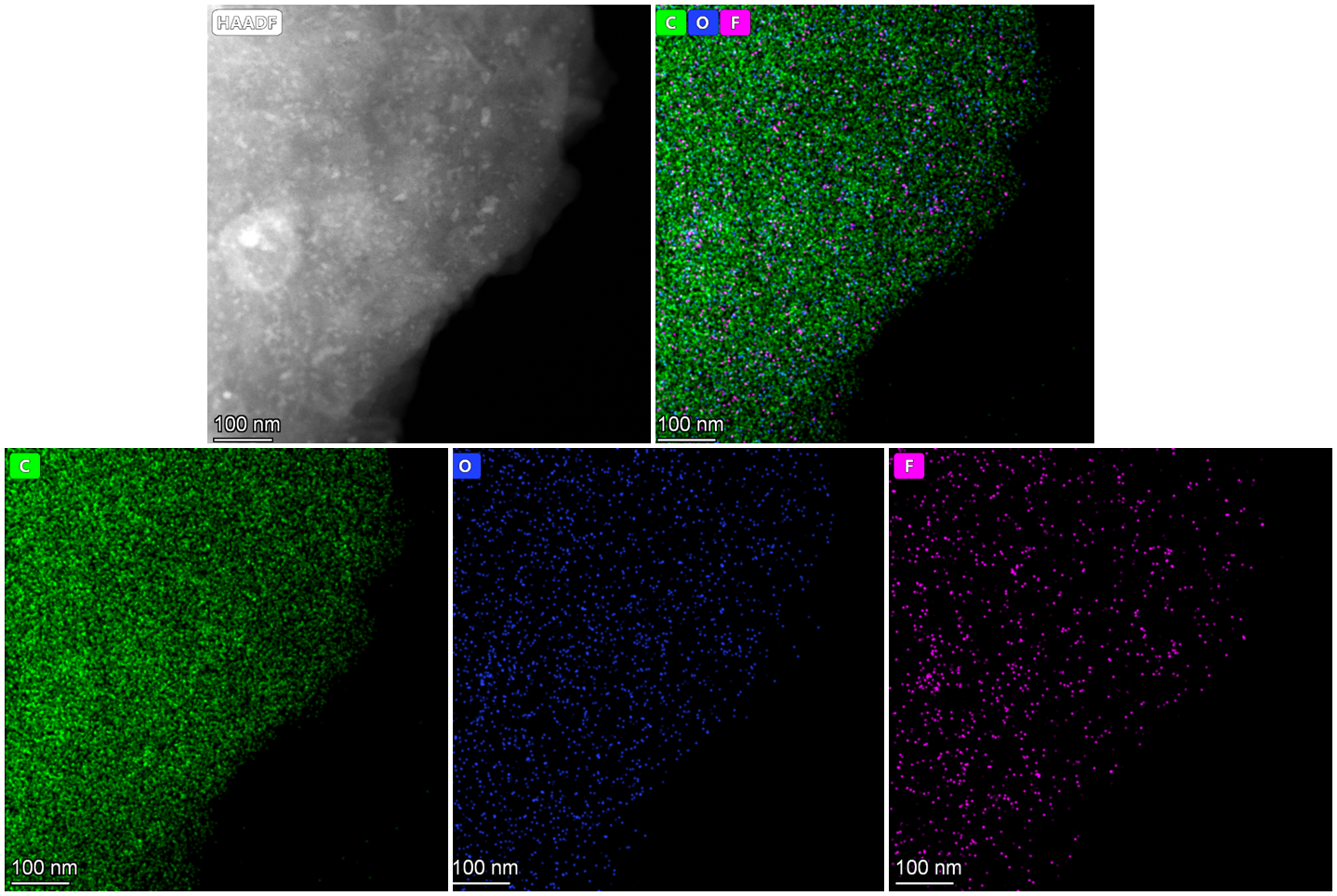


**Figure S6** HAADF-STEM image and energy-dispersive spectroscopy (EDS) mapping of F-GNR. Green, blue, and magenta colors indicate carbon, oxygen, and fluorine, respectively.

**
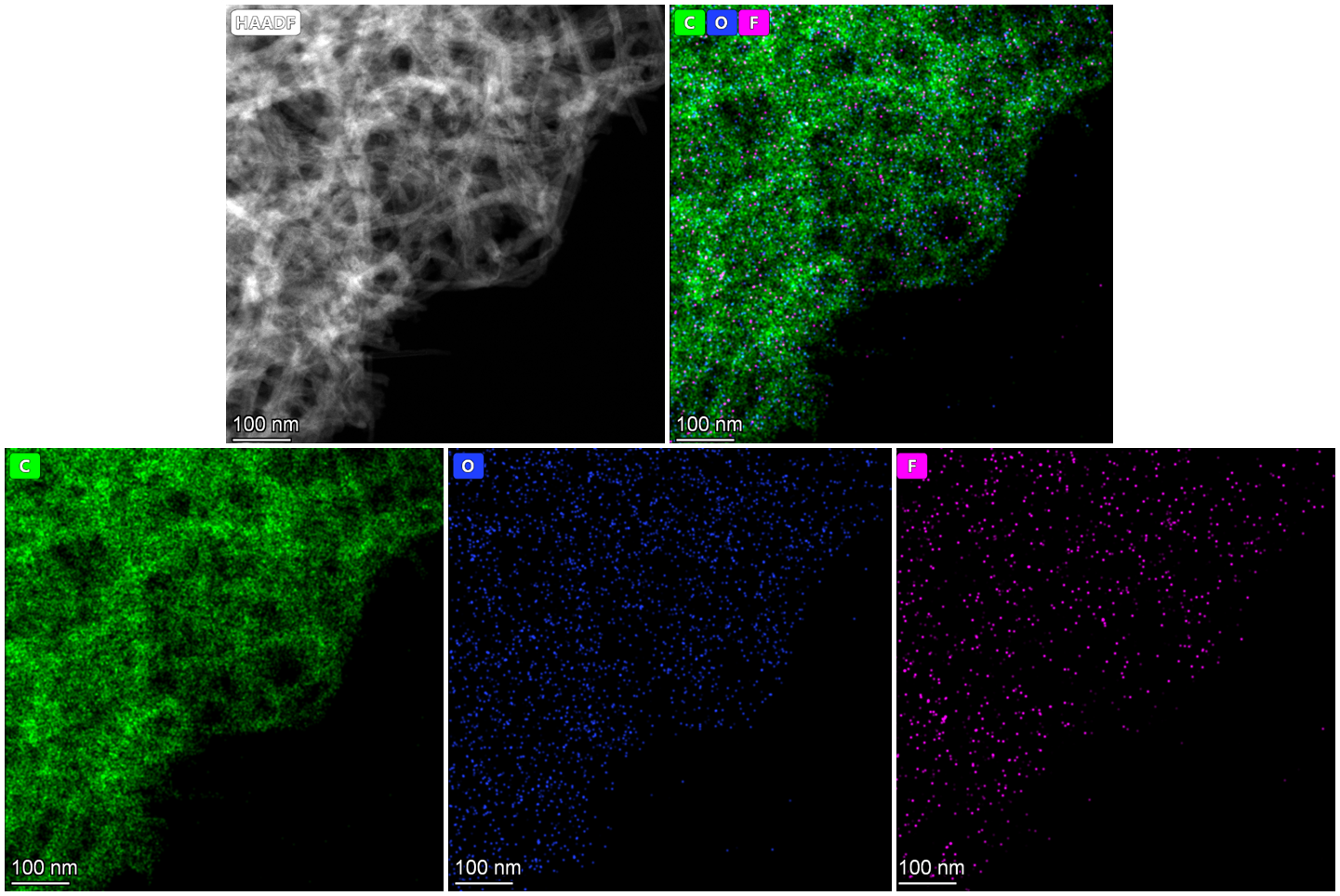
**

**Figure S7** HAADF-STEM image and energy-dispersive spectroscopy (EDS) mapping of F-GNR@CNT focusing on the basal plane. Green, blue, and magenta colors indicate carbon, oxygen, and fluorine, respectively.

**
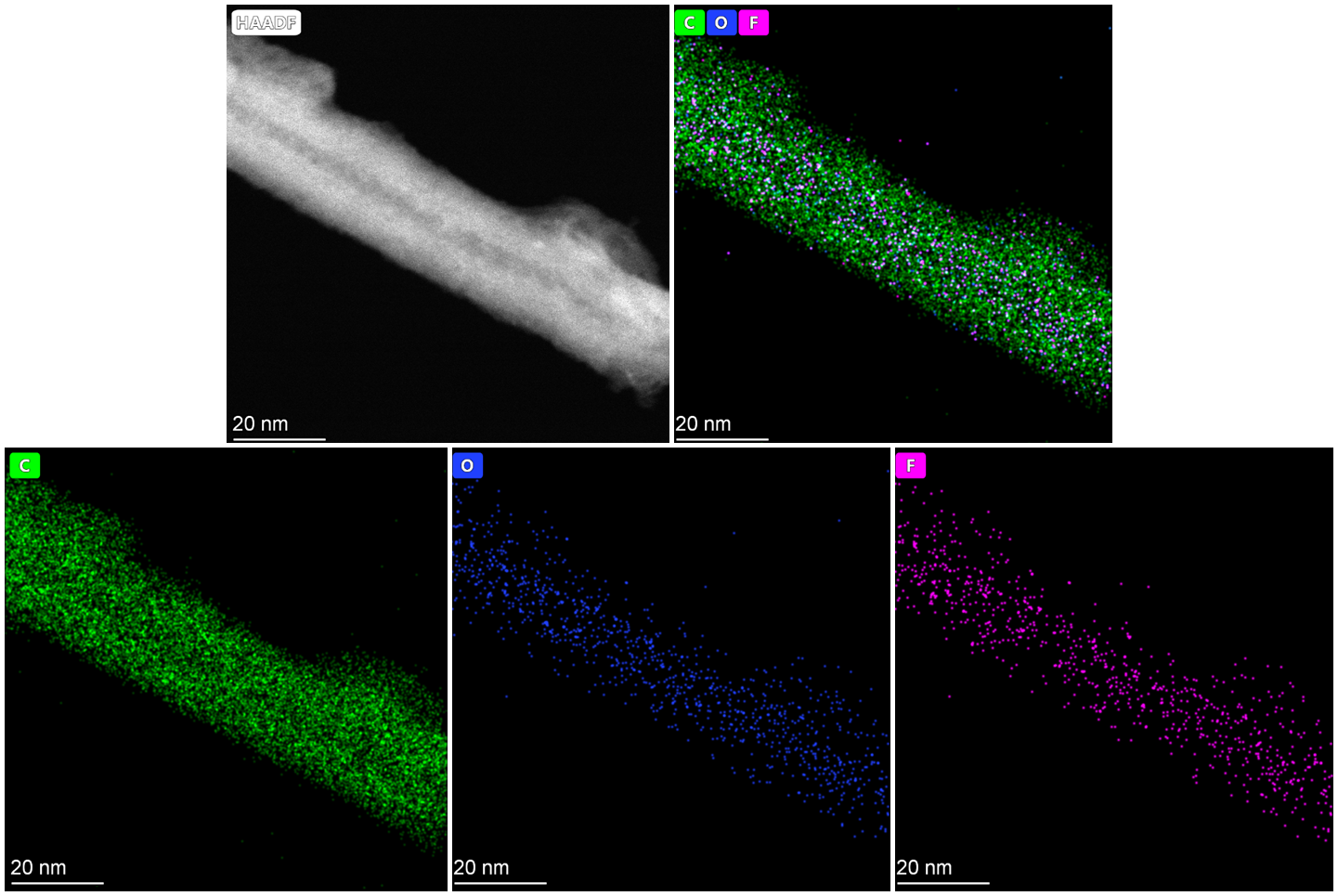
**

**Figure S8** HAADF-STEM image and energy-dispersive spectroscopy (EDS) mapping of F-GNR@CNT in high-resolution focused on having F-CNT features. Green, blue, and magenta colors indicate carbon, oxygen, and fluorine, respectively. **
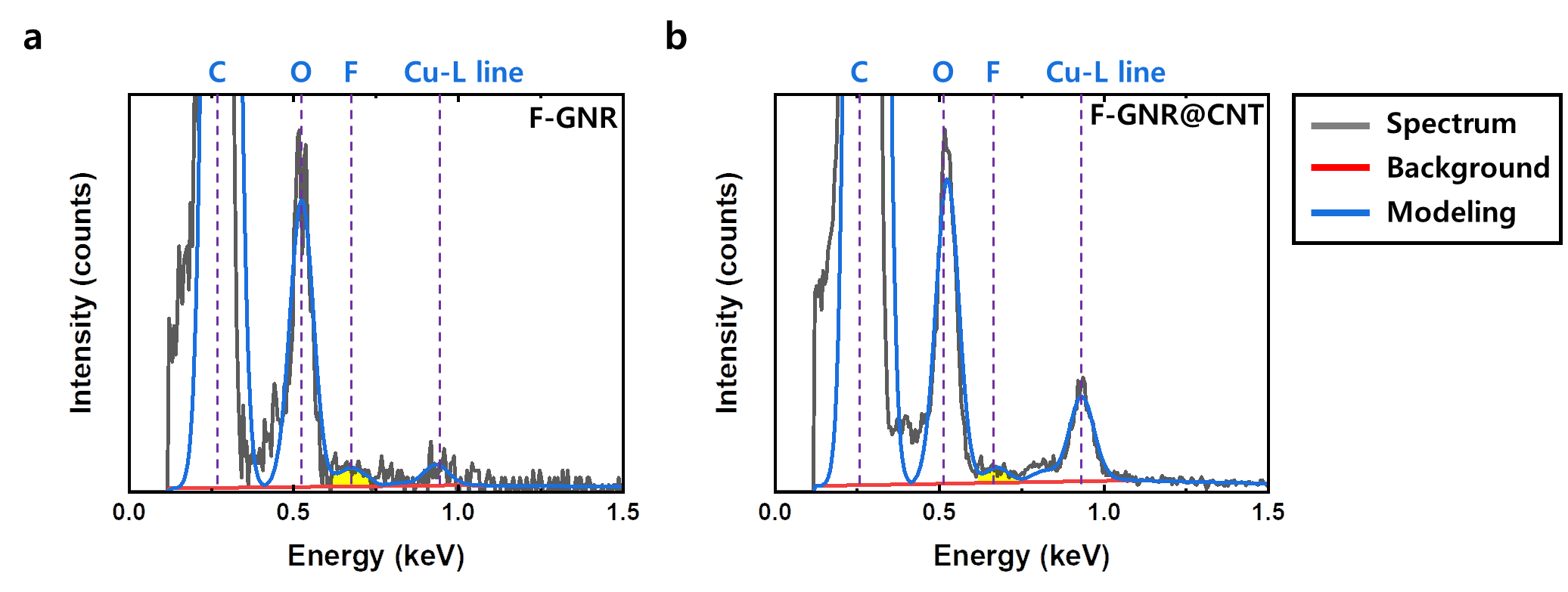
**

**Figure S9** EDS-mapping spectrum and modeling with subtracting background obtained from (a) F-GNR and (b) F-GNR@CNT.


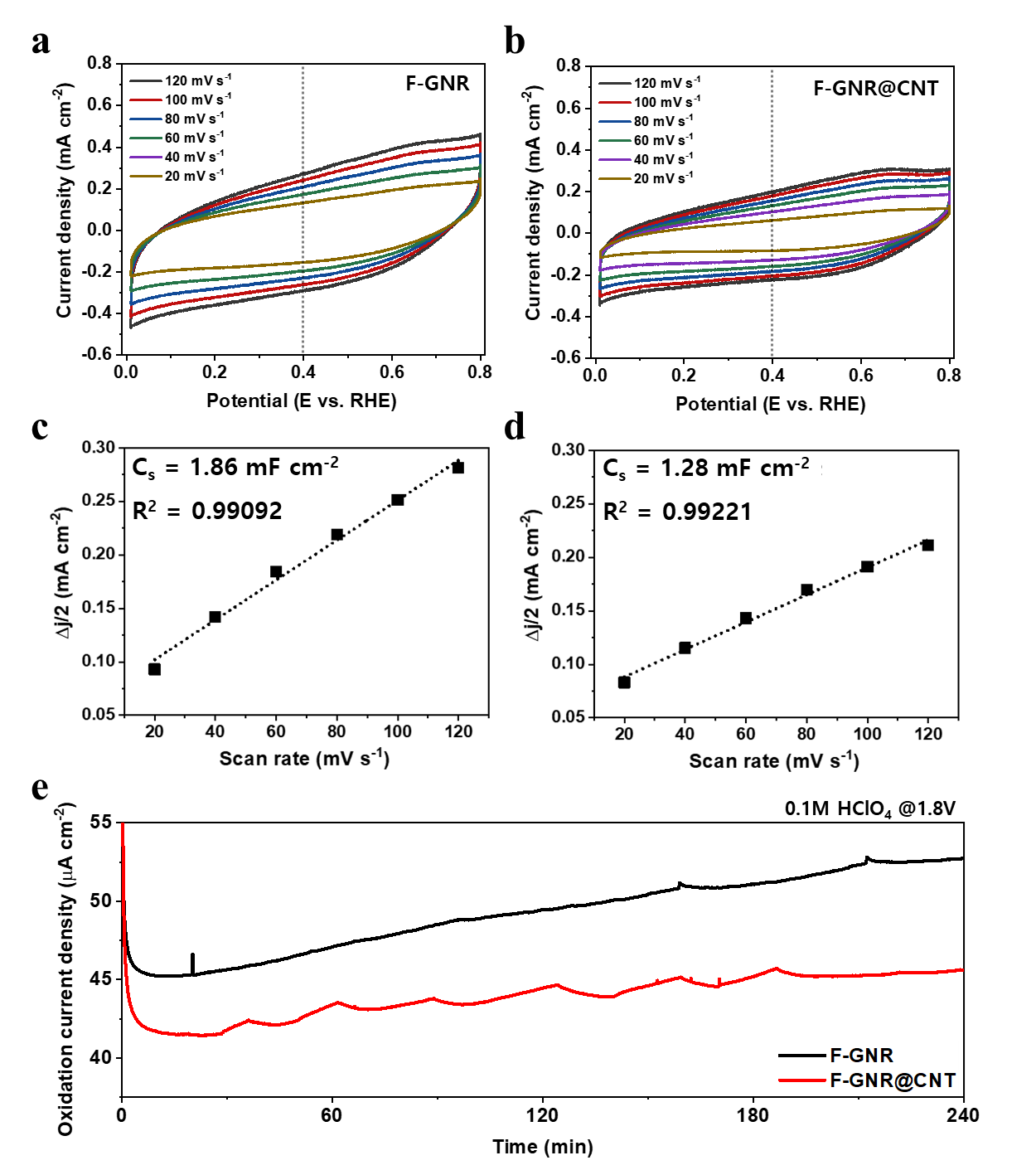


**Figure S10** (a,b) Cyclic voltammograms as a function of scan rate, (c,d) areal capacitance (C_S_) for F-GNR and F-GNR@CNT, respectively.

**Note** This capacitance, characteristic of carbon materials, stores or releases energy via ion adsorption/desorption on the surface under an electric field and applied potential. It is closely linked to the specific surface area and exhibits proportional correlations. To understand and compare the electrochemical surface areas of F-GNR@CNT and F-GNR, the areal capacitance was calculated based on galvanostatic charge-discharge experiments using the following Equation (S1):^[1]^

$C_{s}=I\times t / \Delta U\times S$ (S1)

where C_S_, I, t, U, and S represent the areal capacitance, current (mA), time (s), potential window (V), and working area of the electrode (cm^2^), respectively.


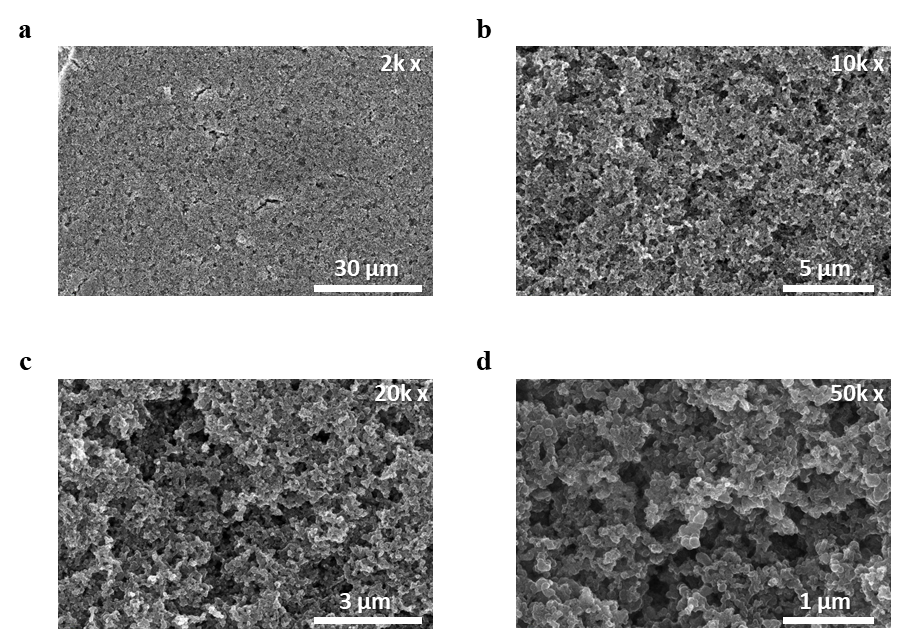


**Figure S11** (a-d) Top view of SEM images for Pt/C cathode from low-resolution (2k) to high-resolution (50k) in order.


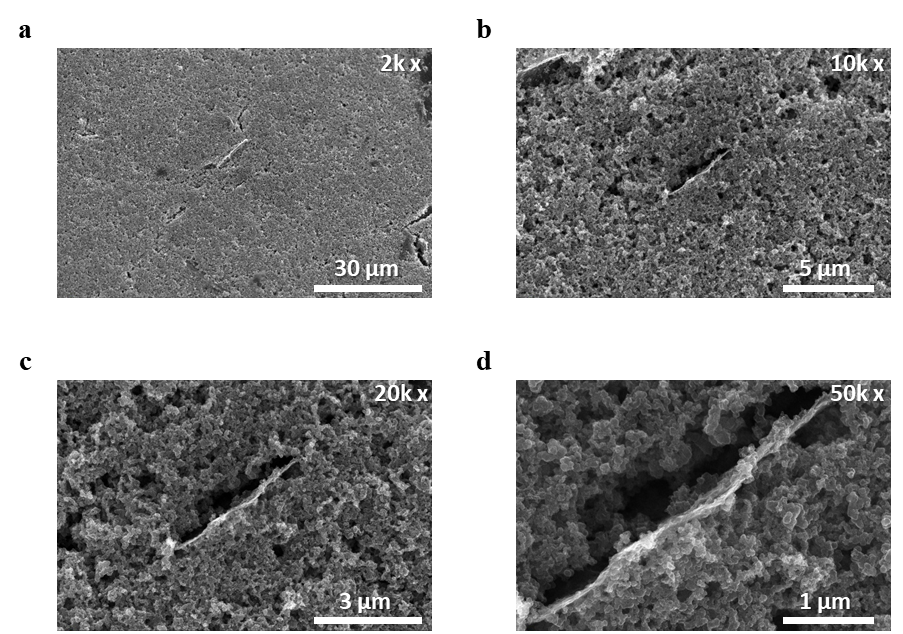


**Figure S12** (a-d) Top view of SEM images for Pt/C + F-GNR cathode from low-resolution (2k) to high-resolution (50k) in order.


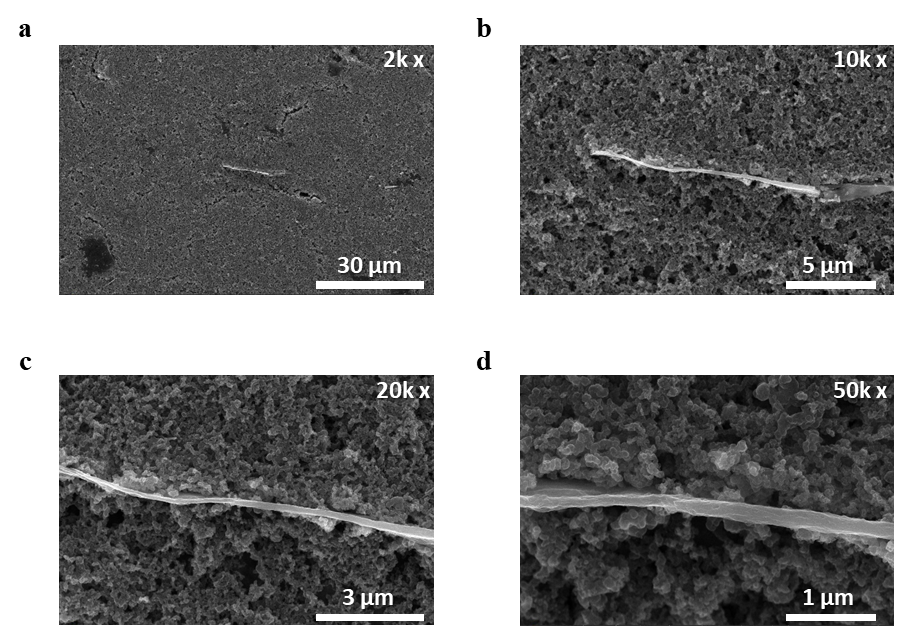


**Figure S13** (a-d) Top view of SEM images for Pt/C + F-GNR@CNT cathode from low-resolution (2k) to high-resolution (50k) in order.


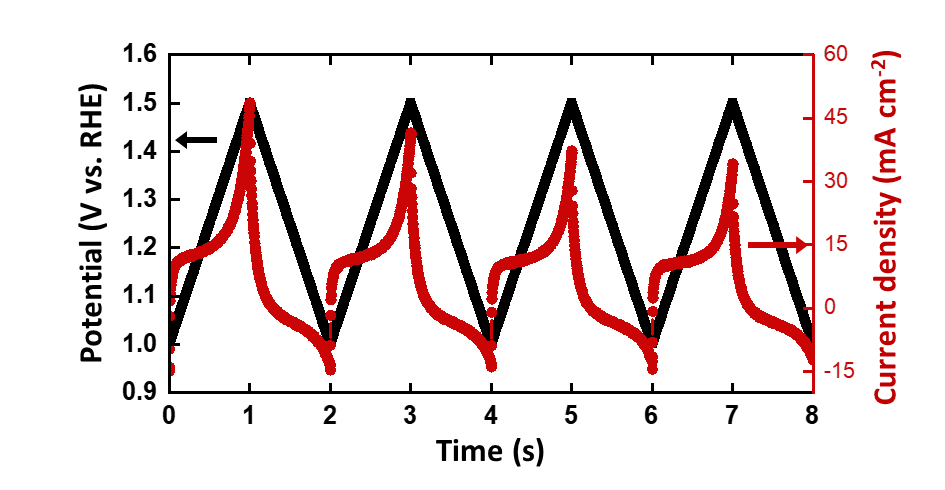


**Figure S14** U.S. DOE protocol for durability test on the carbon corrosion condition. (scan range: 1.0 – 1.5 V vs. RHE; Potential sweep rate: 500 mV s^-1^)


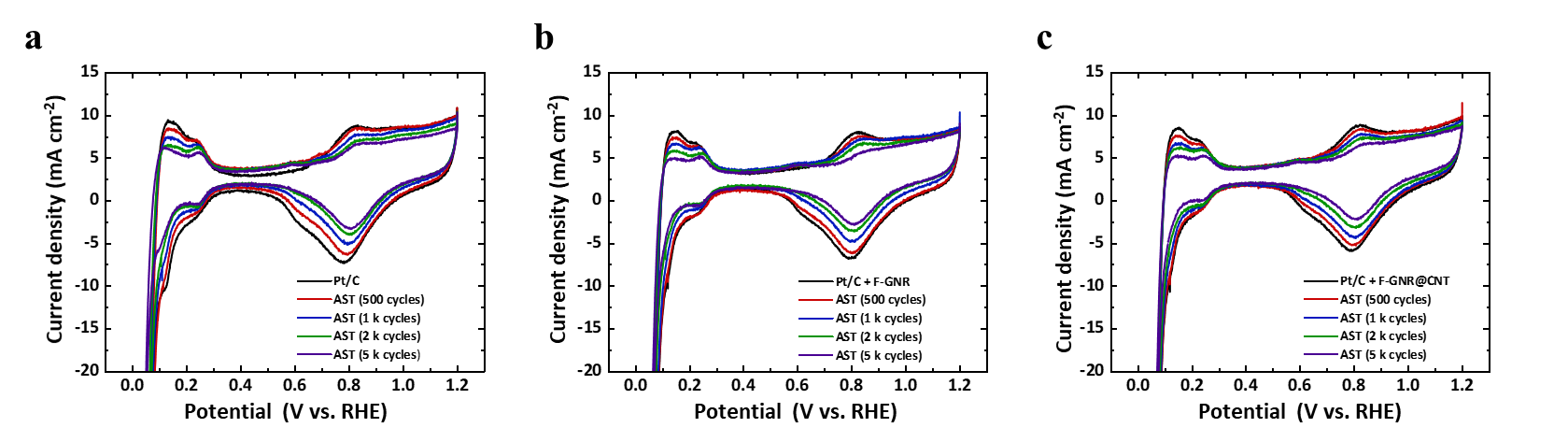


**Figure S15** Cyclic voltammetry plots of (a) Pt/C, (b) Pt/C + F-GNR, and (c) Pt/C + F-GNR@CNT, respectively.

**
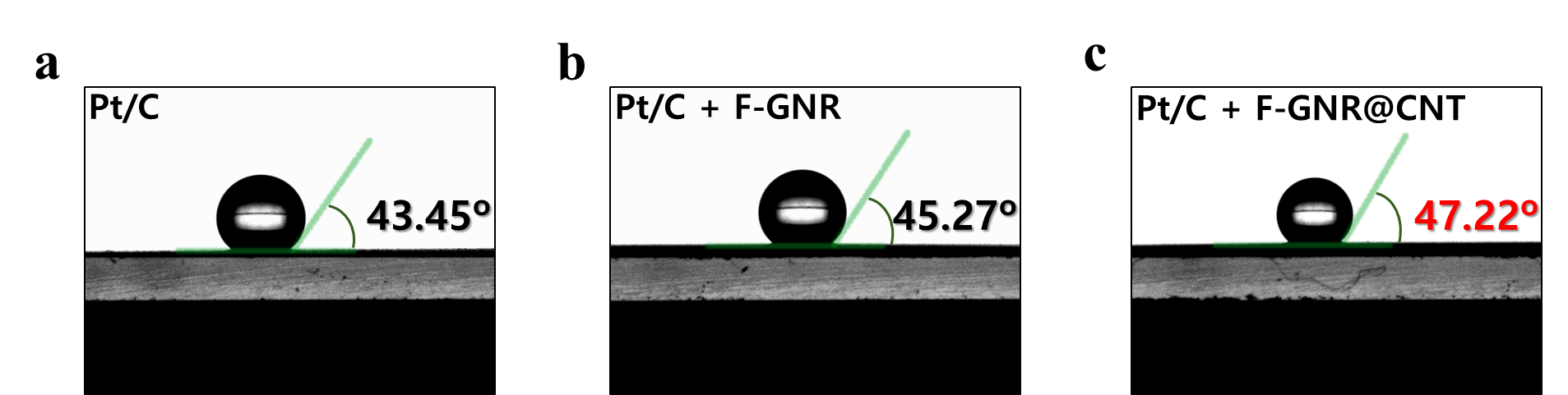
**

**Figure S16** Contact angle images and average degree of (a) Pt/C, (b) Pt/C + F-GNR, and (c) Pt/C + F-GNR@CNT, respectively.


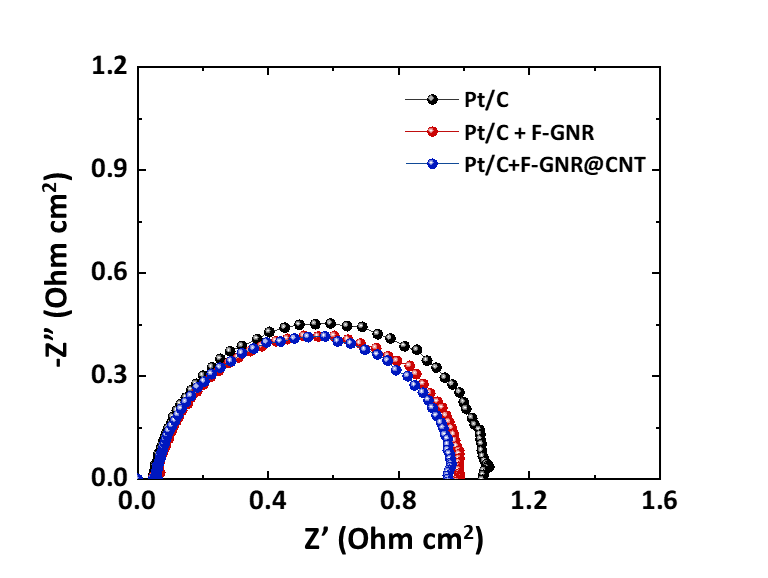


**Figure S17** Galvanostatic electrochemical impedance spectroscopy (GEIS) results measured at a current density of 50 mA cm^-2^ for fresh Pt/C, Pt/C+F-GNR, and Pt/C+F-GNR@CNT electrodes.


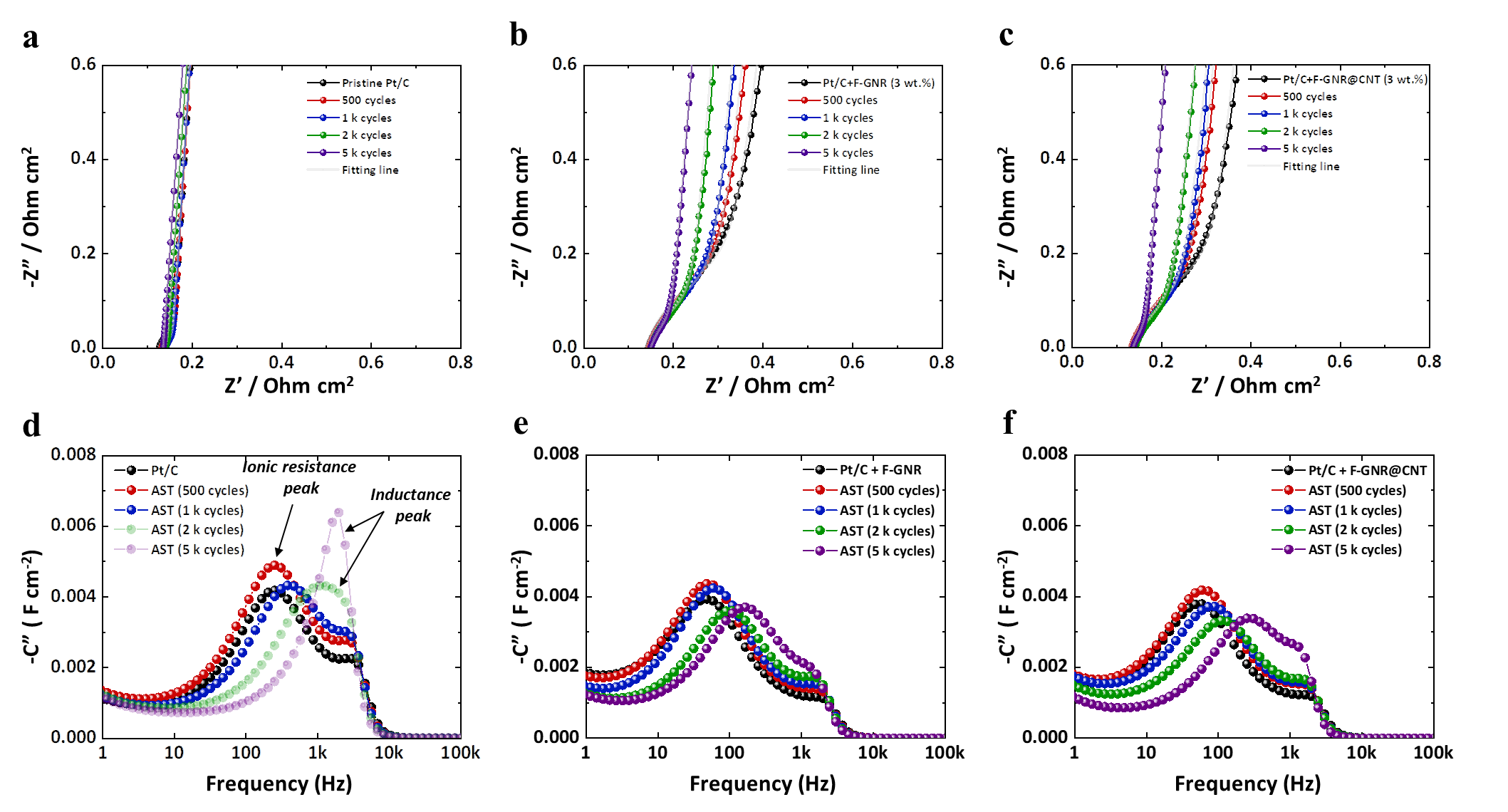


**Figure S18** Non-faradaic impedance results measured at 0.4 V vs. RHE and the fitting lines for degrading (a) Pt/C, (b) Pt/C + F-GNR, and (c) Pt/C + F-GNR@CNT during 5 k of ASTs. The (a-c) results are converted to the complex capacitance, and the (d-f) shows the imaginary capacitance plots of degraded (d) Pt/C, (e) Pt/C + F-GNR, and (f) Pt/C + F-GNR@CNT electrodes, respectively.

**Note**: Numerical analysis for R_ion_ and C_dl_ of degraded electrodes are extracted by fitting processes using Bisquert’s model in *ZView* programs and transmission line model equivalent circuit. The imaginary capacitance plots are converted to complex capacitance plots according to Equation (S2) as follows:

C = $-\frac{Z^{''}}{w\left( Z^{'2}+Z^{''2} \right)}-i\frac{Z^{'}}{w\left( Z^{'2}+Z^{''2} \right)}$ (S2)

, where the second term is the imaginary capacitance plots as shown in Figure S16d-f.

The imaginary capacitance plots could be used to evaluate the variation of capacitive properties of degraded electrodes by carbon corrosion according to the following Equation (S3):

$f_{p}= \frac{0.404}{R_{ion}C_{dl}}$ $\int_{-\infty}^{\infty} C^{''}\left( f \right) dlog\left( f \right)= -0.682 C_{dl}$ (S3)

, where the peak frequency ($f_{p}$) indicates the maximum point at imaginary capacitance plots. Exceptionally, due to the intervention of inductance and negligible value of R_ion_, the capacitive impedance results of Pt/C electrode after 2 k of ASTs show the erroneous capacitance results (marked as inductance peak) in **Figure 19d**. Except for these two cases, all of the numerical values for R_ion_ and C_dl_ are verified by Bode-phase plots during fitting processes and complex capacitance relations.


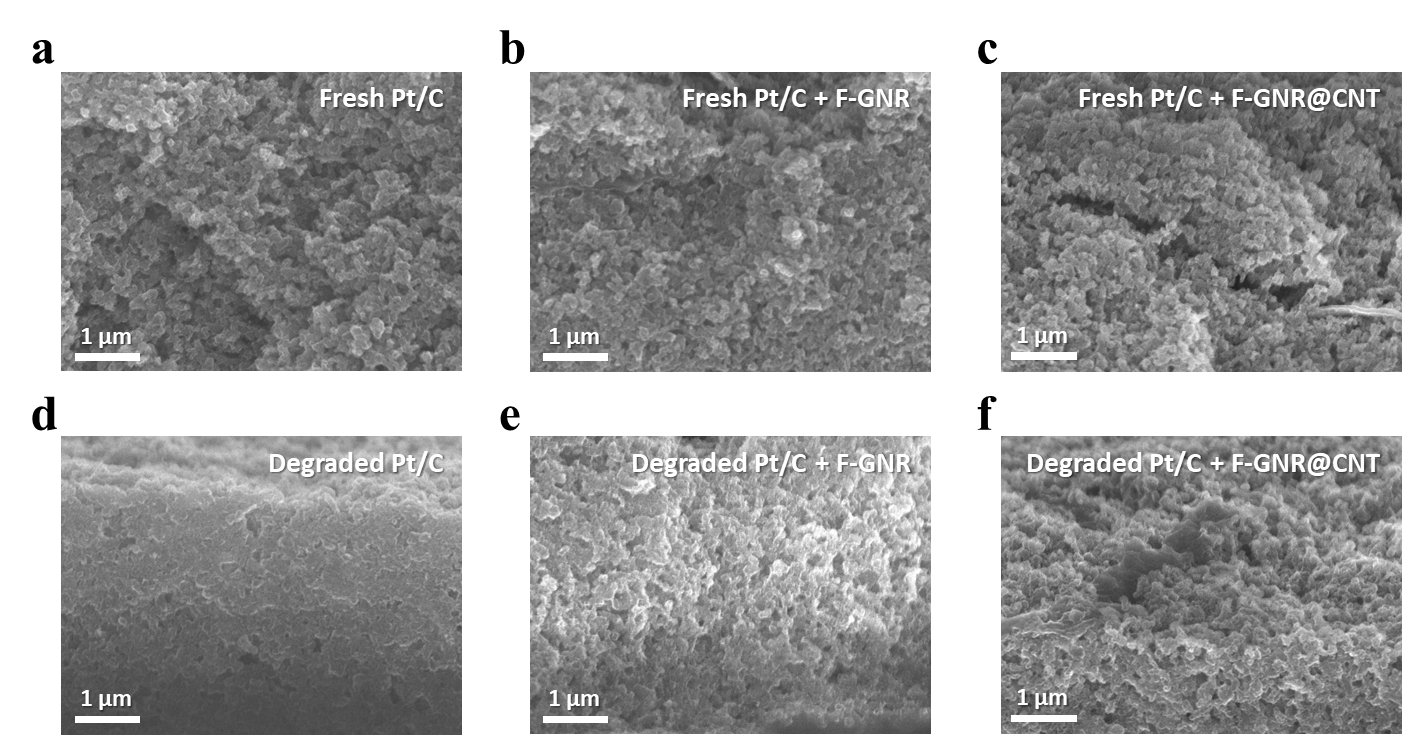


**Figure S19** Fresh and *Post-mortem* SEM images of (a,d) Pt/C, (b,e) Pt/C + F-GNR, and (c,f) Pt/C + F-GNR@CNT, respectively.

**Table S1** Electronegativity comparison of elements on the Pauling scale.^[2]^

| H (2.1) |  |  |  |  |
| --- | --- | --- | --- | --- |
| Li (1.0) | **C (2.5)** | N (3.0) | O (3.5) | **F (4.0)** |
| Na (0.9) | Si (1.8) | P (2.1) | S (2.5) | Cl (3.0) |
| K (0.8) | Ge (1.8) | As (2.0) | Se (2.4) | Br (2.8) |
| Cs (0.7) | Sn (1.8) | Sb (1.9) | Te (2.1) | I (2.5) |

**Table S2** Summary of bond dissociation energies of single covalent bonds with carbon.^[2]^

| Bond | Bond dissociation energy (kJ mol^-1^) |
| --- | --- |
| **C-F** | **485** |
| C-H | 413 |
| C-O | 358 |
| C-C | 348 |
| C-Cl | 328 |
| C-N | 293 |
| C-S | 259 |

**Table S3** Summary of the measured electrical conductivity (S cm^-1^) on various pressure (kgf) for Vulcan carbon, GNRO, F-GNR, GNRO@CNT, and F-GNR@CNT.

|  | **Vulcan carbon** | **GNRO** | **F-GNR** | **GNRO**  **@CNT** | **F-GNR**  **@CNT** |
| --- | --- | --- | --- | --- | --- |
| **Press (kgf)** | **Conductivity (S cm^-1^)** | | | | |
| 199.00 | 5.145 | Not measured | 8.115 | 2.288 | 15.286 |
| 397.50 | 7.781 |  | 10.826 | 2.906 | 19.153 |
| 597.50 | 10.065 |  | 13.004 | 3.455 | 22.156 |
| 797.50 | 12.192 |  | 14.951 | 4.058 | 24.968 |
| 994.50 | 14.187 |  | 16.751 | 4.741 | 27.734 |
| 1194.50 | 16.194 |  | 18.854 | 5.520 | 30.702 |
| 1394.50 | 18.409 |  | 21.140 | 6.532 | 34.229 |
| 1595.00 | 20.757 |  | 23.964 | 7.902 | 38.145 |
| 1794.50 | 23.375 |  | 27.209 | 9.879 | 42.761 |
| 1994.50 | 26.212 |  | 31.226 | 12.525 | 47.680 |

**Table S4** Summary of ECSA value and current density at 0.6V and 0.4V before/after AST (500, 1k, 2k, 5k cycles) for Pt/C, Pt/C + F-GNR, and Pt/C + F-GNR@CNT, respectively.

| **Pt/C** | **ECSA (m^2^ g^-1^)** | **Current density (mA cm^-2^)** | |
| --- | --- | --- | --- |
|  |  | **@ 0.6V** | **@ 0.4V** |
| Pristine | 36.68 | 714.89 | 1815.6 |
| 500 cycles | 29.58 | 393.89 | 1803.3 |
| 1 k cycles | 24.06 | 267.33 | 1517.8 |
| 2 k cycles | 20.43 | 291.22 | 704.67 |
| 5 k cycles | 17.78 | **120.89** | **265.11** |
| **Pt/C + F-GNR** | **ECSA (m^2^ g^-1^)** | **Current density (mA cm^-2^)** | |
|  |  | **@ 0.6V** | **@ 0.6V** |
| Pristine | 29.01 | 1029.1 | 1791.1 |
| 500 cycles | 25.12 | 916.33 | 1765.6 |
| 1 k cycles | 20.62 | 805.33 | 1722.2 |
| 2 k cycles | 18.68 | 517.22 | 1601.1 |
| 5 k cycles | 14.41 | **327.33** | **983.78** |
| **Pt/C + F-GNR@CNT** | **ECSA (m^2^ g^-1^)** | **Current density (mA cm^-2^)** | |
|  |  | **@ 0.6V** | **@ 0.6V** |
| Pristine | 27.11 | 943.22 | 1733.3 |
| 500 cycles | 21.73 | 934.22 | 1737.8 |
| 1 k cycles | 19.34 | 881.00 | 1668.9 |
| 2 k cycles | 17.87 | 680.44 | 1525.6 |
| 5 k cycles | 11.61 | 375.89 | 1038.3 |

**Table S5** Summary of internal resistance and capacitance values of degraded electrode during 500, 1 k, 2 k, and 5 k of AST cycles for Pt/C, Pt/C + F-GNR, and Pt/C + F-GNR@CNT, respectively.

| **Pt/C** | **R_ct_ at 50 mA cm^-2^ (Ohm cm^2^)** | **Capacitive impedance results (30% RH condition)** | | | | |
| --- | --- | --- | --- | --- | --- | --- |
|  |  | **HFR(Ohm cm^2^)** | | **R_ion_ (Ohm cm^2^)** | | **C_dl_ (F cm^-2^)** |
| Pristine | 1.00 | 0.104 | 0.158 | | 0.0128 | |
| 500 cycles | 1.67 | 0.113 | 0.135 | | 0.0149 | |
| 1 k cycles | 1.73 | 0.122 | 0.104 | | 0.0130 | |
| 2 k cycles | 2.06 | 0.123 | 0.063 | | 0.0122 | |
| 5 k cycles | 2.96 | 0.123 | 0.036 | | 0.0111 | |
| **Pt/C + F-GNR** | **R_ct_ at 50 mA cm^-2^ (Ohm cm^2^)** | **Capacitive impedance results (30% RH condition)** | | | | |
|  |  | **HFR(Ohm cm^2^)** | | **R_ion_ (Ohm cm^2^)** | | **C_dl_ (F cm^-2^)** |
| Pristine | 0.93 | 0.072 | 0.765 | | 0.0133 | |
| 500 cycles | 0.93 | 0.077 | 0.675 | | 0.0141 | |
| 1 k cycles | 0.98 | 0.086 | 0.603 | | 0.0139 | |
| 2 k cycles | 1.29 | 0.095 | 0.432 | | 0.0113 | |
| 5 k cycles | 1.81 | 0.104 | 0.270 | | 0.0113 | |
| **Pt/C +**  **F-GNR@CNT** | **R_ct_ at 50 mA cm^-2^ (Ohm cm^2^)** | **Capacitive impedance results (30% RH condition)** | | | | |
|  |  | **HFR(Ohm cm^2^)** | | **R_ion_ (Ohm cm^2^)** | | **C_dl_ (F cm^-2^)** |
| Pristine | 0.90 | 0.070 | 0.720 | | 0.0123 | |
| 500 cycles | 0.89 | 0.070 | 0.576 | | 0.0137 | |
| 1 k cycles | 0.92 | 0.080 | 0.486 | | 0.0127 | |
| 2 k cycles | 1.03 | 0.084 | 0.405 | | 0.0111 | |
| 5 k cycles | 1.42 | 0.093 | 0.216 | | 0.0106 | |

**References**

[1] Z. H. Huang, T. Y. Liu, Y. Song, Y. Li, X. X. Liu, *Nanoscale* **2017**, 9 (35), 13119

[2] D. O'Hagan, *Chem. Soc. Rev.* **2008,** 37, 308–319
